# Ionic Charge-Transfer Liquid Crystals Formed by Alternating Supramolecular Copolymerization of Liquid π-Donors and TCNQ

**DOI:** 10.3389/fchem.2021.657246

**Published:** 2021-03-29

**Authors:** Hiroaki Iguchi, Hidenori Furutani, Nobuo Kimizuka

**Affiliations:** ^1^Department of Chemistry and Biochemistry, Graduate School of Engineering, Kyushu University, Fukuoka, Japan; ^2^Department of Chemistry, Graduate School of Science, Tohoku University, Sendai, Japan; ^3^Center for Molecular Systems (CMS), Kyushu University, Fukuoka, Japan

**Keywords:** ionic charge-transfer complex, liquid donors, liquid crystals, supramolecular alternate copolymerization, self-assembly

## Abstract

A new family of liquid π-donors, lipophilic dihydrophenazine (DHP) derivatives, show remarkably high π-electron-donor property which exhibit supramolecular alternating copolymerization with 7,7,8,8-tetracyanoquinodimethane (TCNQ), giving ionic charge-transfer (ICT) complexes. The ICT complexes form distinct columnar liquid crystalline (LC) mesophases with well-defined alternating molecular alignment as demonstrated by UV-Vis-NIR spectra, IR spectra, and X-ray diffraction (XRD) patterns. These liquid crystalline ICT complexes display unique phase transitions in response to mechanical stress: the columnar ICT phase is converted to macroscopically oriented smectic-like mesophases upon applying shear force. Although there exist reports on the formation of ICT in the crystalline state, this study provides the first rational identification of ICT mesophases based on the spectroscopic and structural data. The liquid crystalline ICT phases are generated by strong electronic interactions between the liquid π-donors and solid acceptors. It clearly shows the significance of simultaneous fulfillment of strong π-donating ability and ordered self-assembly of the stable ICT pairs. The flexible, stimuli-responsive structural transformation of the ICT complexes offer a new perspective for designing processable CT systems with controlled hierarchical self-assembly and electronic structures.

## Introduction

Ionic CT (ICT) complexes have been receiving great interest from chemists, physicists, and materials scientists because of their attractive electronic properties. The first significant breakthrough in ICT complexes is the discovery of the first metallic conduction in tetrathiafulvalene/7,7,8,8-tetracyanoquinodimethane (TTF-TCNQ) (Ferraris et al., 1973). Its high electrical conductivity originates from the segregated alignment of the ionized donor (D) and acceptor (A) molecules (Kistenmacher et al., 1974). These findings have led to the development of related materials and applications (Frére and Skabara, 2005; Pauliukaite et al., 2007; Piek et al., 2018; Fujihara et al., 2020). On the other hand, another type of ICT complexes, i.e., those consist of alternately stacked arrays of the ionized D and A molecules, have recently been attracted attention because they are expected to serve as potential platforms for designing advanced materials such as organic ferroelectrics (Horiuchi and Tokura, 2008; Kobayashi et al., 2012; Takehara et al., 2019), multiferroics (Kagawa et al., 2010; Wang and Zhang, 2020), and photovoltaics (Nakamura et al., 2017). In contrast to the conventional neutral CT complexes, reports on ICT complexes have been largely limited to crystalline solids. Although room-temperature ferroelectricity has been reported for crystalline supramolecular ICT complexes (Tayi et al., 2012), they are currently regarded as neutral CT complexes and the suggested effect of crystal defects made the CT-ferroelectricity relationship indecisive (D'Avino et al., 2017; Tayi et al., 2017; Chen et al., 2019). Not limited to this issue, crystals, in general, are not suitable for applying ICT characteristics for developing useful devices that require the control of molecular orientations continuously from the molecular to macroscopic scales. It is also desirable that the ICT complexes show dynamic self-assembly and self-healing properties so that their physical properties are determined without the serious influence of crystalline defects. These problems will be solved by developing soft, self-assembling ICT complex systems. Although the CT interaction has been used to prepare columnar liquid crystals (Ringsdorf et al., 1989; Wang et al., 2009; Das and Ghosh, 2014; Bé et al., 2015) and other supramolecular nanostructures (Han et al., 2018; Adelizzi et al., 2019), their classes of CT are mostly neutral or undefined. To date, only two columnar liquid crystals have been reported as ICT complexes. However, they fall short of the critical requirements, that is, the 1:1 stoichiometry of DA pairs (Stepień et al., 2007) and the evidence for alternate DA stacking (Saeva et al., 1982; Stepień et al., 2007). The difficulty arises from the lack of rational methodologies to obtain the actual ICT state and to secure the regular, supramolecular alternating alignment of the ionized D and A molecules in soft molecular self-assemblies.

To develop ICT complexes that show self-assembly properties, we designed alternating supramolecular copolymerization of liquid donors and a benchmark acceptor TCNQ. To form ICT complexes with the 1:1 stoichiometry, it is essential that the donors have significantly strong electron-donating properties. In addition, the donors and acceptors are required to select only alternate supramolecular copolymerization into linear ICT complexes without segregation of each component. In order to achieve such an alternating molecular arrangement in soft molecular systems, where stabilization by the lattice force in 3D crystalline solids is not available, at least one component molecule is required to have an adaptive property so that the other component can be accommodated in between them by allowing the structural mismatch. In this work, we focused on engineering donor molecules because it is more facile than modifying acceptor molecules. To meet the above requirements, we adopted room-temperature liquefaction of π-donors by introducing proper substituents because such π-donor liquids are expected to show a limited degree of intermolecular interactions between identical molecules. This feature is also advantageous to solubilize the low-molecular-weight π-acceptors and achieve adaptive self-assembly, thereby leading to the ordered, alternating supramolecular copolymerization of ionized donors and acceptors. The room-temperature π-liquid became widely known for octyl methoxycinnamate, which has been utilized as an ultraviolet absorbing agent over several decades (Marti-Mestres et al., 2000; Scalia and Mezzena, 2010). It was popularized by the development of liquid chromophores containing carbazole (Hendrickx et al., 1999; Hirata et al., 2011), anthracene (Babu et al., 2013; Duan et al., 2013), oligo (*p*-phenylene vinylene) (Babu et al., 2012), azobenzene (Masutani et al., 2014), and so forth (Snaith et al., 2006; Kamino et al., 2012; Li et al., 2013; Takeda et al., 2018; Ghosh et al., 2019; Isoda et al., 2019; Lu and Nakanishi, 2019; Ogoshi et al., 2019; Morikawa et al., 2020; Bai et al., 2021). These π-liquids have been utilized as liquid semiconductors (Hendrickx et al., 1999; Snaith et al., 2006; Hirata et al., 2011; Kamino et al., 2012; Li et al., 2013), solvent-free luminescent liquids (Babu et al., 2012, 2013; Lu and Nakanishi, 2019; Bai et al., 2021), stimuli-responsive liquid (Takeda et al., 2018; Isoda et al., 2019; Ogoshi et al., 2019), liquid electret (Ghosh et al., 2019), photon upconverters (Duan et al., 2013), and solar thermal fuels (Masutani et al., 2014; Morikawa et al., 2020). Although some π-conjugated organic liquids reported to date show electron-donating or accepting properties, they are categorized as weak donors or acceptors (Babu and Nakanishi, 2013; Lu and Nakanishi, 2019). The CT complexes are classified by the degree of CT (ρ_CT_), where ρ_CT_ = 0 for neutral TCNQ and ρ_CT_ = 1 for complete charge transfer as found for K-TCNQ (Chappell et al., 1981). The vast majority of existing CT complexes reported to date fall into the class of neutral CT complexes with smaller ρ_CT_ values (0 < ρ_CT_ < 0.5), and only those having large ρ_CT_ values (0.5 < ρ_CT_ ≤ 1) are referred to as ICT complexes. The neutral to ionic transition of CT complexes has been induced by applying pressure (Torrance et al., 1981b; Takehara et al., 2019) or at a considerably lower temperature of 81 K for a tetrathiafluvalne-*p*-chloranil complex (Torrance et al., 1981a; Okamoto et al., 1991). These conditions severely limit the applicability of ICT complexes for device applications. There exist no reports on the strong donor or acceptor π-liquids that can reduce the counterpart molecules to give ICT complexes under ambient conditions, and no general strategy has been developed to obtain ICT complexes without applying high pressure or low-temperature conditions. Meanwhile, we have previously reported that CT interactions among one-dimensional cationic mixed-valence platinum complexes are significantly enhanced by the integration with regularly aligned anionic synthetic lipid molecules (Kimizuka, 2000). It is thus reasonable that the introduction of the suitable molecular design cultivated by self-assembly research to solid-state ICT complexes would provide a new perspective to design functional, soft ICT complexes.

**Scheme 1:**
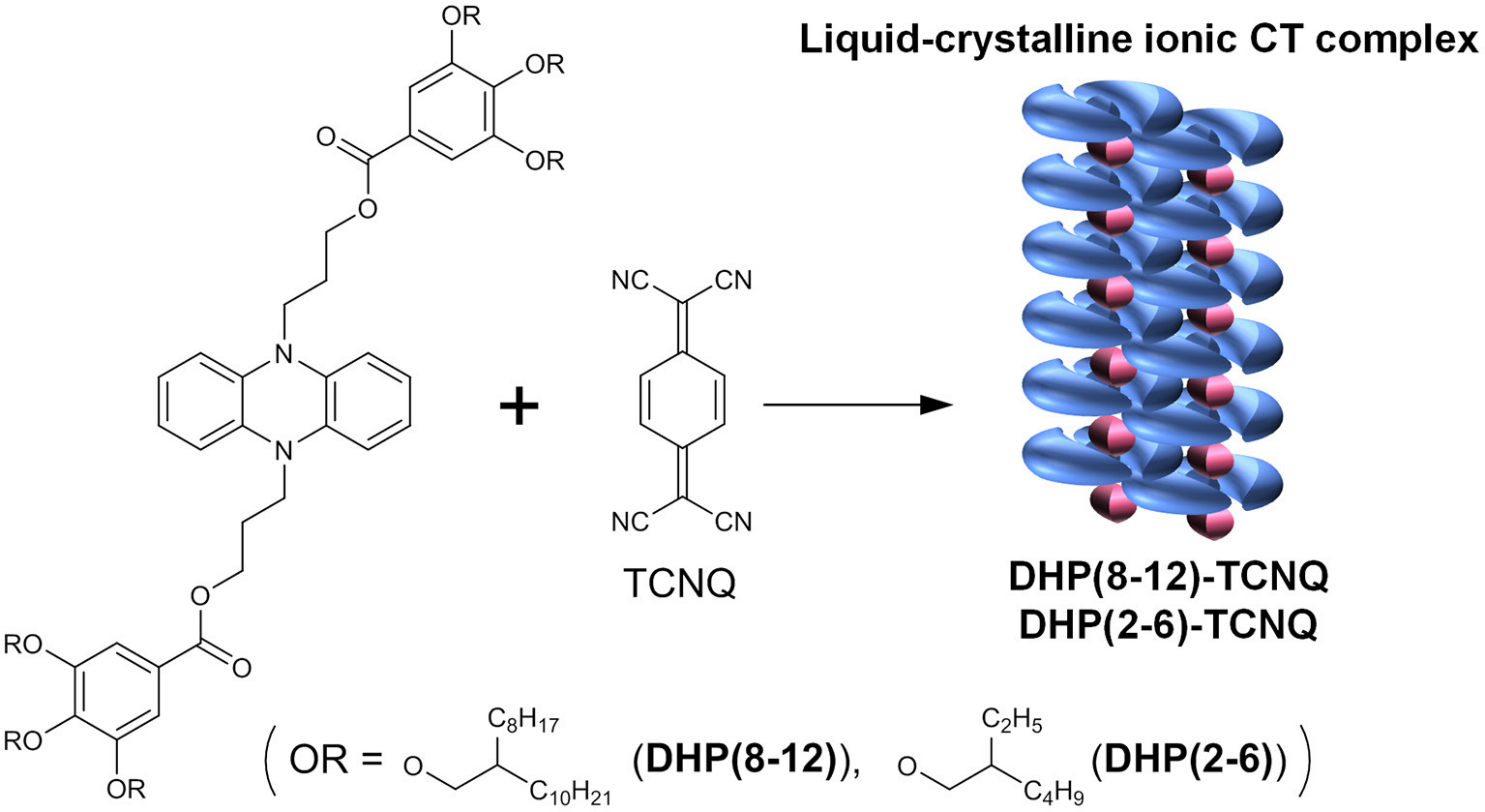
Liquid-crystalline ionic CT complexes self-assembled from DHP derivatives and TCNQ.

Herein, we describe a rational self-assembly approach to obtain well-defined, alternately stacked liquid crystalline ICT complexes. π-Conjugated room-temperature liquids of dihydrophenazine (DHP) derivatives that show the highest electron-donating ability ever were developed. Moderately bulky branched alkyl substituents are linked to the DHP units via spacer alkyl units, which molecular design not only increases the solubility but also creates a flexible void in between the π-donor molecules, allowing adaptive accommodation of acceptors in the condensed state. By simply mixing equimolar liquid-DHP and TCNQ in solution and casting at ambient temperature, alternating supramolecular copolymerization facilely occurs into ICT complexes in which ICT complexes are linearly aligned in the core of liquid crystals (Scheme 1). The self-assembly of liquid donor and crystalline acceptor gives rise to a new form of liquid crystalline ICT complexes, which feature has been demonstrated for the first time. These ICT liquid crystals displayed physical stimuli-responsive dynamic structural transformation, which characteristics affords us opportunities to control their molecular alignment hierarchically from the nano-to-macroscopic scales.

## Results and Discussion

### Preparation and Electronic State of ICT Complexes

As a donor chromophore, DHP was selected since the pair of 5,10-dihydro-5,10-dimethylphenazine [DHP(Me)] and TCNQ, a strong acceptor, has been reported to give a crystalline CT complex with a relatively large ρ_CT_ value (ρ_CT_ = 0.5 ± 0.1) (Meneghetti et al., 1985), which indicates that the complex is with intermediate ionicity. The DHP chromophore was liquefied according to the strategy to reduce cohesive interactions, i.e., by introducing branched alkyl groups (2-octyldodecyl and 2-ethylhexyl substituents) into 3,4,5-trialkoxyphenyl periphery groups of the donor chromophore DHP [**DHP(8-12)** and **DHP(2-6)**, respectively] (Scheme 1), since we expected that these fluid and bulky side chains would promote solubilization and provide an adaptive space that accommodates TCNQ molecules without segregation. The length of branched alkyl chains in **DHP(8-12)** and **DHP(2-6)** was changed in order to tune their intermolecular interactions and guest-accommodating properties.

**Figure 1:**
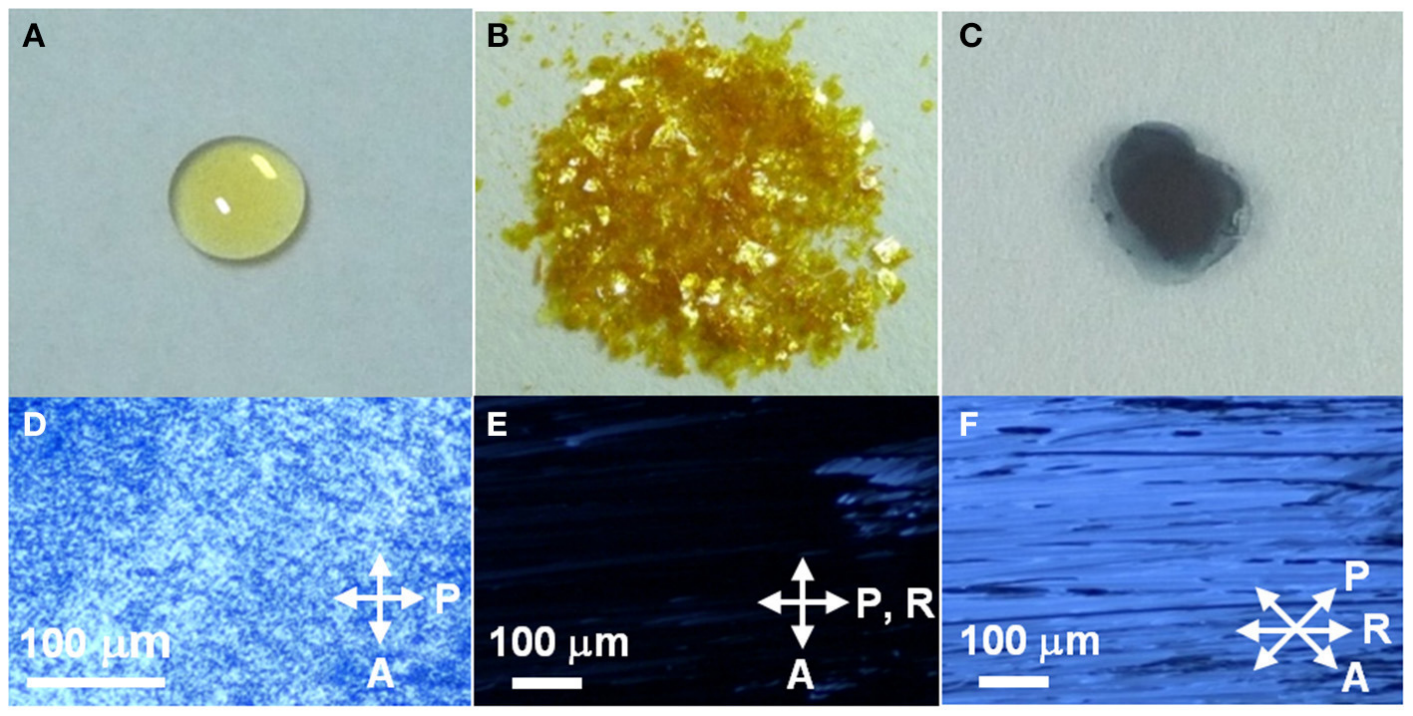
Photographs of **(A) DHP(8-12)**, **(B)** TCNQ, **(C) DHP(8-12)-TCNQ**, and **(D–F)** optical micrographs of the cast films of **DHP(8-12)-TCNQ** between crossed polarizer (P) and analyzer (A) for different orientations of the sample, given by the angle θ between the rubbing direction (R) and P. **(D)** as cast film, **(E)** after rubbing, θ = 0°, **(F)** θ = 45°. Arrows indicate the directions of the analyzer (A), a polarizer (P), and rubbing (R).

**Figure 2:**
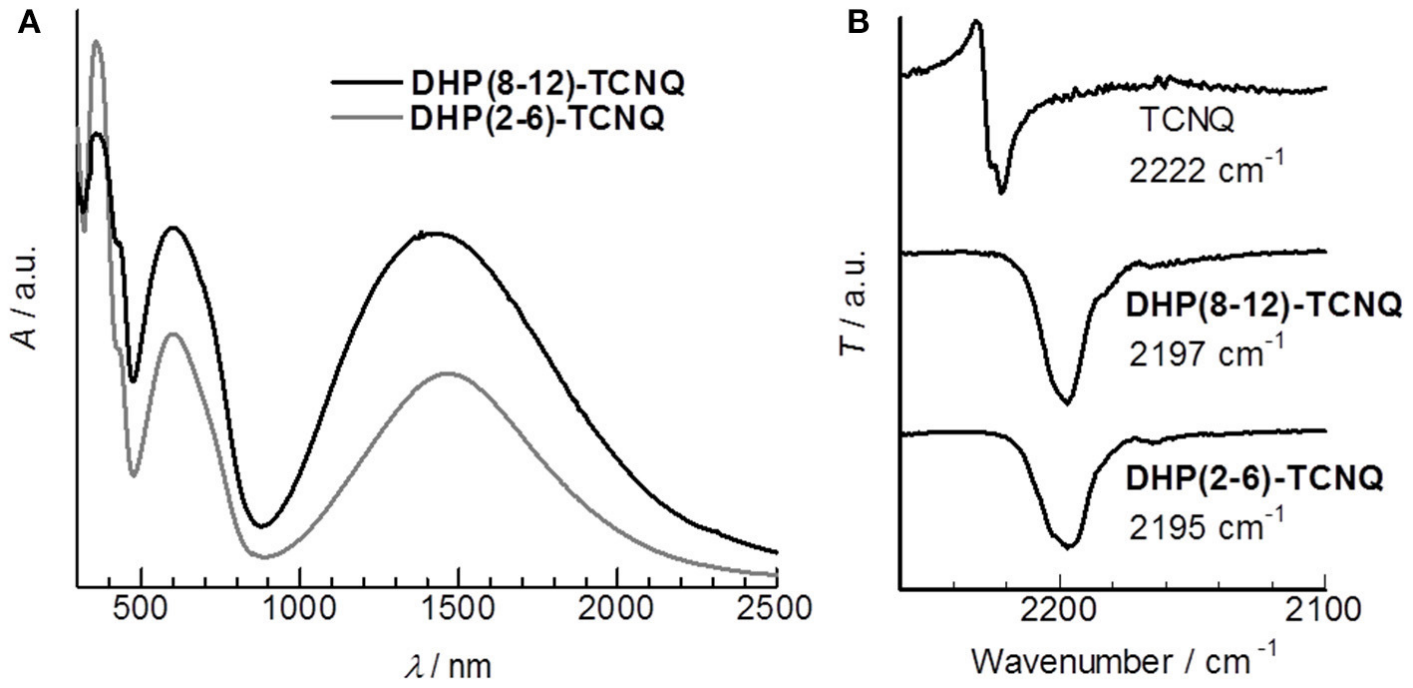
**(A)** UV-Vis-NIR spectra of the cast film of **DHP(8-12)-TCNQ** (black) and **DHP(2-6)-TCNQ** (gray). **(B)** IR spectra of TCNQ, **DHP(8-12)-TCNQ**, and **DHP(2-6)-TCNQ** with each wavenumber value for C≡N stretching vibration (ν_C≡N_).

As expected, these DHP derivatives were obtained as isotropic, pale yellow liquids at room temperature (RT) (Figure 1A). The viscosity of the liquid was comparable to that of liquid paraffin. Interestingly, a significantly low first oxidation potential [$E_{1}^{◦x}$ = −0.23 V (vs. Fc/Fc^+^)] was observed in the cyclic voltammogram of **DHP(2-6)** (Supplementary Figure 1). It is lower than that of the reported π-donor liquids [e.g., $E_{1}^{◦x}$ = +0.17 V (vs. Fc/Fc^+^) for tris-[4-(2-methoxyethoxy)-phenyl]-amine (Snaith et al., 2006)] and that of the representative donor, TTF [$E_{1}^{◦x}$ = −0.10 V (vs. Fc/Fc^+^) (Keniley et al., 2013)], providing an unequivocal basis that these DHP derivatives fall into the category of the strongest π-conjugated electron-donor liquid. Since $E_{1}^{◦x}$ is comparable to the first reduction potential for TCNQ [$E_{1}^{\text{red}}$ = −0.25 V (vs. Fc/Fc^+^)] (Endo et al., 2014), the formation of ICT complexes is expected from the pair of DHP derivatives and TCNQ. To prepare CT complexes of **DHP(8-12)-TCNQ** and **DHP(2-6)-TCNQ**, equimolar toluene solutions of DHP derivatives and TCNQ (Figure 1B) were mixed. Interestingly, upon casting these solutions on quartz substrates, deep blue films (Figure 1C) were obtained at RT. The absorption spectra of these films show broad peaks at 1422 nm (0.872 eV) for **DHP(8-12)-TCNQ** and at 1467 nm (0.845 eV) for **DHP(2-6)-TCNQ**, respectively (Figure 2A). These peaks are reasonably assignable to the ICT absorption bands by reference to that reported for the DHP(Me)-TCNQ crystal (0.84–0.89 eV) (Fujita and Matsunaga, 1980; Soo et al., 1981). It is to note that additional peaks are observed around at 360 nm (3.44 eV) with a shoulder component around 430 nm (2.88 eV) and at 600 nm (2.07 eV), which are also reported for DHP(Me)-TCNQ (3.41 eV, 2.85 eV and 1.98 eV) (Fujita and Matsunaga, 1980). These peaks are assignable to the intramolecular electronic transitions for anion and cation radicals formed in the ICT complexes, as referenced to the previous reports on K-TCNQ (3.17–3.39 eV and 2.00 eV) (Torrance et al., 1980; Ikegami et al., 2007) and DHP(Me)-PF_6_ (3.3 eV and 2.6 eV) (Ohkura et al., 2011). The ρ_CT_ values of these CT complexes can be determined based on the linear correlation between the charge of TCNQ and the frequency of its C≡N stretching vibration (ν_C≡N_) (Chappell et al., 1981). In IR spectra (Figure 2B), the ν_C≡N_ was observed at 2197 cm^−1^ for **DHP(8-12)-TCNQ** and 2195 cm^−1^ for **DHP(2-6)-TCNQ**, while a neutral TCNQ (ρ_CT_ = 0) gave the peak at 2222 cm^−1^. As K-TCNQ (ρ_CT_ = 1) was reported to give a ν_C≡N_ peak at 2183 cm^−1^ (Chappell et al., 1981), ρ_CT_ values for **DHP(8-12)-TCNQ** and **DHP(2-6)-TCNQ** are determined as 0.64 and 0.69, respectively (see details in the Supplementary Material). These ρ_CT_ values are sufficiently large to conclude their ICT characteristics.

### Liquid crystalline formation by ICT complexes

Figures 1D–F shows optical micrographs of the cast films of **DHP(8-12)-TCNQ** prepared on glass slides. The cast film under crossed polarizers [polarizer axis (P) ⊥ analyzer axis (A)] showed light transmission due to birefringence revealed by the anisotropic liquid crystalline domains (Figure 1D). Very interestingly, although the cast film is highly viscous, these domains underwent fusion and alignment by applying mechanical shear force (rubbing treatment). As shown in Figure 1E, a “dark” state with very little birefringence was observed when the sample was rubbed along the direction of the polarizer axis P (P || R). On the other hand, when P is 45° from the rubbing direction (R), a bright image was observed with an anisotropic texture oriented along with R (Figure 1F). Similar results were also obtained for the cast film of **DHP(2-6)-TCNQ** (Supplementary Figures 2B,C). These observations clearly indicate that the ICT complexes are in the liquid crystalline phase, which is distinct from the liquid and crystalline phase revealed by each donor and acceptor component, respectively. It is noteworthy that the in-plane macroscopic orientation of these liquid crystalline ICT complexes is controllable by applying mechanical force.

### Structural Evidence for the Alternate Stacking

**Figure 3:**
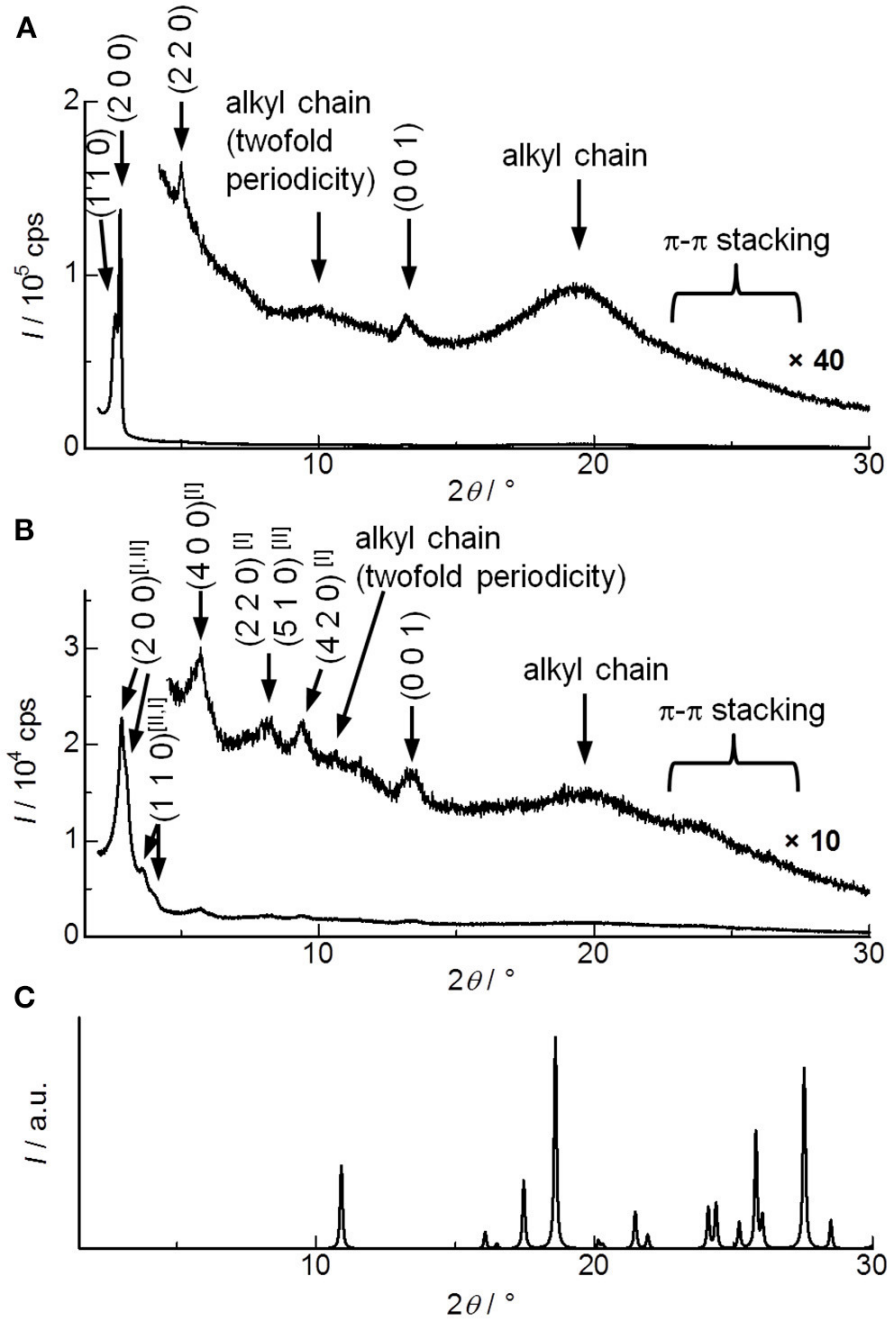
XRD patterns for **(A) DHP(8-12)-TCNQ**, **(B) DHP(2-6)-TCNQ**, and **(C)** TCNQ (simulated from the reported crystal structure) (Long et al., 1965). Magnified figures of **DHP(8-12)-TCNQ** and **DHP(2-6)-TCNQ** in low angle region (2–5°) are shown in the insets of Figure 6 (gray line). Superscript [I] and [II] presented with the Miller indices indicate two distinct phases assigned in Table 1.

In order to analyze molecular alignments in these liquid crystalline ICT complexes, X-ray diffraction (XRD) measurements were conducted for the as-cast films. Figure 3A shows the XRD patterns obtained for **DHP(8-12)-TCNQ**. Strong diffractions are observed in the small-angle region, which can be assigned as (110) and (200) reflections of columnar rectangular (Col_r_) liquid crystalline phases (*a* = 63.3 Å, *b* = 41.5 Å, *c* = 6.72 Å). Detailed data and assignments are shown in Table 1. The broad halos around 10 and 20° are ascribed to reflections from the liquid-like cluster of branched alkyl chains. Importantly, a distinct peak is observed at 13.16° (6.72 Å), which is assignable to a (001) reflection as the spacing coincides with the two-fold distance of typical π-π stacking (3.2–3.4 Å). It indicates the presence of highly ordered, periodical alignment of DHP molecules in the alternately stacked columnar DHP-TCNQ complex structures. Such a reflection from two-fold periodicity has not been commonly observed (Tsukruk et al., 1993; Park et al., 2003; Bé et al., 2015). Although it has been observed for a few columnar liquid crystals of non-ionic CT complexes (Ringsdorf et al., 1989; Das and Ghosh, 2014), the present UV-VIS, IR spectroscopy, and XRD data indicate that the self-assembly of **DHP(8-12)** with TCNQ form liquid crystalline ICT complexes.

**Table 1:** XRD data for cast films of **DHP(8-12)-TCNQ** and **DHP(2-6)-TCNQ**.

	**2θ/^**◦**^^a^**	***d*_**exp**_/Å^b^**	***d*_**theo**_/Å^b^**	**Miller index**
**DHP(8-12)-TCNQ**	2.57	34.3	34.7	(1 1 0)
Col_r_	2.79	31.6	31.6	(2 0 0)
*a* = 63.3 Å	5.00	17.7	17.4	(2 2 0)
*b* = 41.5 Å	9.96 br	8.87		alkyl chain^c^
*c* = 6.72 Å	13.16	6.72	6.72	(0 0 1)
*V* = 17653 Å^3^	19.3 br	4.59		alkyl chain
ρ = 0.94 g cm^−3^	23**–**27	3.9**–**3.3		π-π stacking
*Z* = 4				
**DHP(2-6)-TCNQ**	2.82	31.3	31.3	(2 0 0) phase I
Phase I, Col_r_	2.97	29.7	29.7	(2 0 0) phase II
*a* = 62.6 Å	3.59	24.6	24.6	(1 1 0) phase II
*b* = 23.2 Å	4.06	21.7	21.7	(1 1 0) phase I
*c* = 6.64 Å	5.70	15.5	15.7	(4 0 0) phase I
*V* = 9637 Å^3^	8.18	10.8	10.9	(2 2 0) phase I
ρ = 1.02 g cm^−3^				(5 1 0) phase II
*Z* = 4	9.37	9.43	9.31	(4 2 0) phase I
Phase II, Col_r_	10 br	8.8		alkyl chain^c^
*a* = 59.5 Å	13.3	6.64	6.64	(0 0 1)
*b* = 27.1 Å	19.6 br	4.52		alkyl chain
*c* = 6.64 Å	23**–**27	3.9**–**3.3		π-π stacking
*V* = 10684 Å^3^				
ρ = 0.92 g cm^−3^				
*Z* = 4				

a *Characteristics of the reflections: br, broad*.

b *d_exp_ and d_theo_ are the experimentally measured and theoretical diffraction spacings, respectively*.

c *Reflecting two-fold periodicity*.

The structural regularity of ICT complexes in the Col_r_ phase and the absence of phase-separated TCNQ is further supported by the absence of reflections assignable to segregated TCNQ crystals (Figure 3C). Although reflections derived from the π-π stacking of chromophores in typical columnar liquid crystals appear around 23–27°, they are also not observed in the present **DHP(8-12)-TCNQ** system. It confirms that the stacking of identical **DHP(8-12)** chromophores is interrupted by the TCNQ molecules located in between them. The small lattice constant *c* (6.72 Å) observed for the Col_r_ liquid crystalline phase of **DHP(8-12)-TCNQ** suggests that the central aromatic chromophores are oriented almost parallel to the *ab*-plane, i.e., with nearly perpendicular orientation with respect to the column axes.

In the case of **DHP(2-6)-TCNQ**, XRD patterns similar to those for **DHP(8-12)-TCNQ** were observed (Figure 3B; Table 1). However, they reveal two coexisting Col_r_ liquid crystalline phases. Highly ordered alternate stacking is also achieved in **DHP(2-6)-TCNQ**, which shows (001) reflection at 13.3° (6.64 Å).

**Figure 4:**
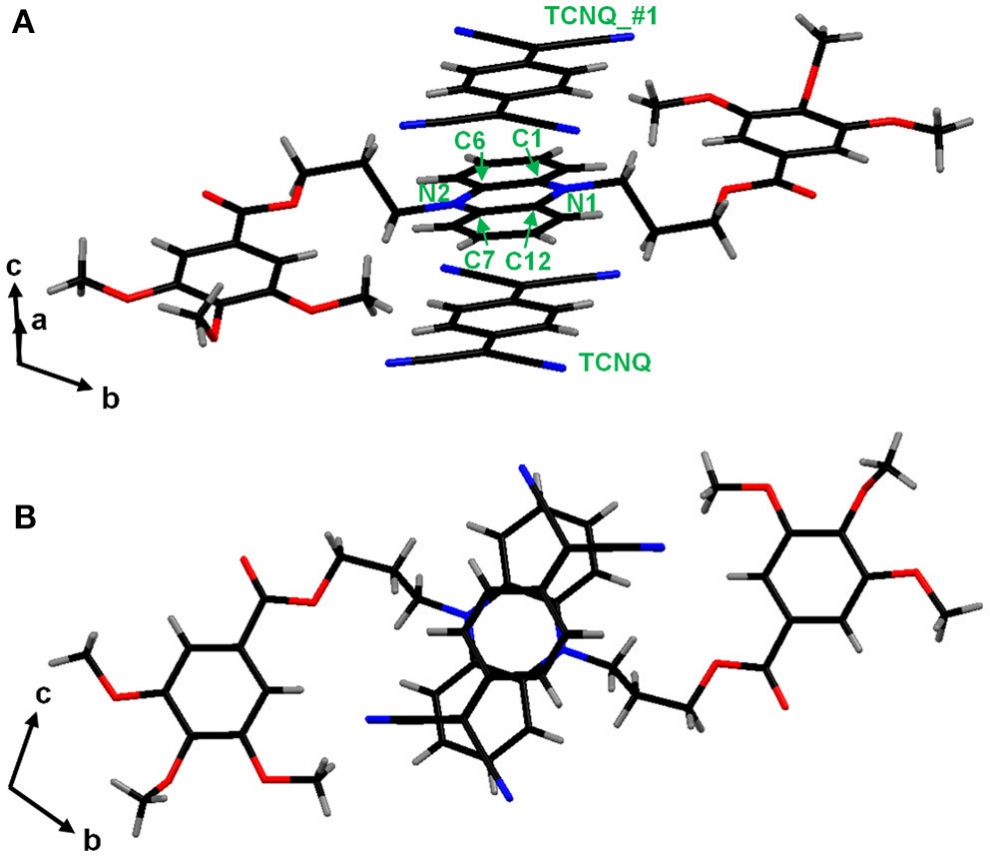
π-π stacking structure of **DHP1-TCNQ·2CH**_**3**_**CN (A)** with atomic and molecular labels mentioned in Supplementary Table 2, and **(B)** viewed from a-axis. Symmetry transformations used to generate equivalent atoms: #1: *x* + 1, *y, z*.

To get further insight into the structure of ICT complex, single-crystalline CT complex was obtained from a crystalline DHP derivative (**DHP1**), which has methoxy substituents instead of the branched alkyl groups, and TCNQ. The single-crystal X-ray structural analysis of **DHP1-TCNQ·2CH**_**3**_**CN** (Supplementary Table 1) clearly shows that **DHP1** and TCNQ stack alternately, as shown in Figure 4. In addition, the TCNQ molecule is not at the midpoint between two **DHP1** molecules but located closer to either one of the two, which is the characteristic of polar ICT complexes (Supplementary Table 2). The crystalline **DHP1-TCNQ·2CH**_**3**_**CN** showed IR spectral characteristics similar to those observed in liquid crystalline ICT complexes. As shown in Supplementary Figure 4, the peaks at 2203 cm^−1^ and 2188 cm^−1^ are assignable to b_1u_ and a_g_ modes of ν_C≡N_ in the TCNQ molecule, respectively (Meneghetti et al., 1985). The b_1u_ mode, which is unclear in the liquid crystalline ICT complexes, is well-identified due to the high regularity in the single crystal. The ρ_CT_ value for **DHP1-TCNQ·2CH**_**3**_**CN** calculated from a_g_ modes is 0.87, which indicates that the compound is in the ICT state. These data support that the liquid crystalline **DHP(2-6)-TCNQ** and **DHP(8-12)-TCNQ** also form alternate stacking with the ICT state, whereas their inter-column packing structures should be different from that of **DHP1-TCNQ·2CH**_**3**_**CN** crystal.

### Packing Structure for Liquid Crystalline ICT Complexes

**Figure 5:**
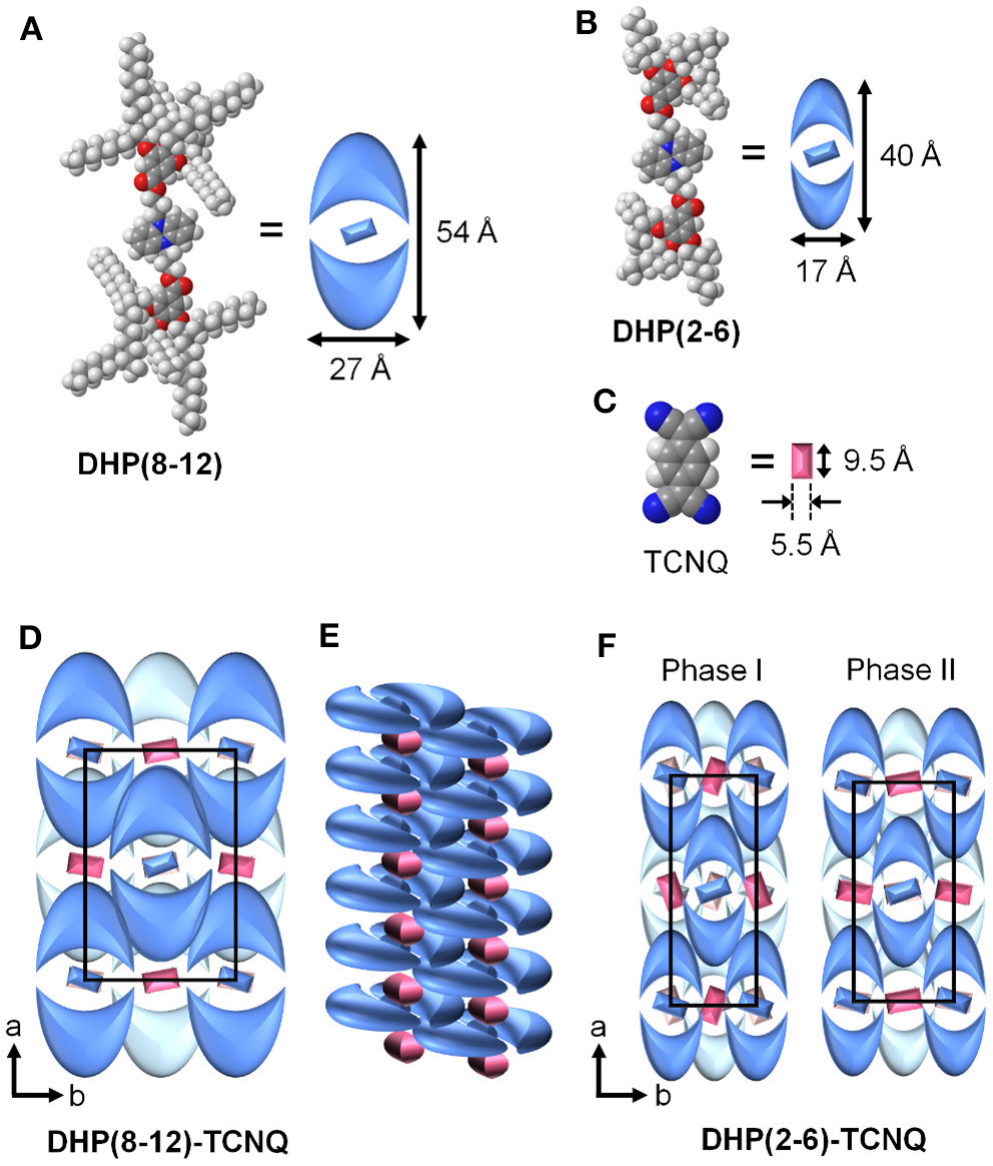
Schematic representation of **(A) DHP(8-12)**, **(B) DHP(2-6)**, **(C)** TCNQ. **(D)** Estimated packing model for **DHP(8-12)-TCNQ** viewed from the column axis (*c*-axis). Molecules on the back layer are shown in a lighter color. **(E)** Schematic packing model for neighbor columns of **DHP(8-12)-TCNQ**, showing that the alkyl chains are penetrated each other. **(F)** Estimated packing model for **DHP(2-6)-TCNQ** viewed from the column axis (*c*-axis).

The molecular packing model of **DHP(8-12)-TCNQ** and **DHP(2-6)-TCNQ** can be determined by the above information from XRD patterns. As can be seen from the CPK model (Figures 5A–C), **DHP(8-12)** possesses an oval disks structure with its long axis oriented parallel to the *a*-axis (Figure 5D). The size of oval discs was roughly estimated by considering the motion of liquid-like alkyl chains and their entanglement with those of the neighboring molecules (Figures 5D,E). This packing model would fit the face-centered orthorhombic lattice if these liquid crystals possessed 3D long-range ordering. Since the number of formula units in the unit cell (*Z*) of this packing model is four, the density (ρ) of the CT complexes is calculated to be 0.94 g cm^−3^ (Table 1). This ρ value is reasonable for organic liquid crystals, supporting the validity of the current supramolecular packing model. The two coexisting liquid crystalline phases in **DHP(2-6)-TCNQ** would be attributed to the presence of bimodal distribution in the molecular orientation of TCNQ as schematically depicted in Figure 5F. The length of lipophilic alkyl chains thus plays an important role in regulating the structural order of columnar ICT liquid crystals.

### Changes in the Macroscopic Orientation by Rubbing Treatment

**Figure 6:**
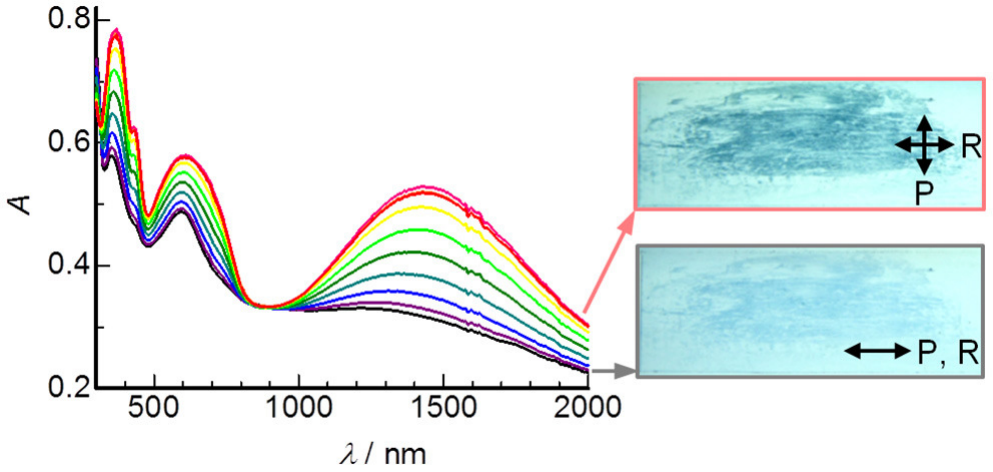
Variations of polarized UV-Vis-NIR spectra of **DHP(8-12)-TCNQ** rubbed film with changing the angle between the polarizer (P) and rubbing (R) direction in steps of 10°. Lower and upper insets show transmission images of the film taken in P || R (black) and in P ⊥ R (red), respectively.

Figure 6 shows polarized UV-Vis-NIR spectra of a unidirectionally rubbed film of **DHP(8-12)-TCNQ** prepared on a quartz substrate, with varying the angle between the polarizer axis (P) and rubbing direction (R). Both the ICT absorption (at 1422 nm) and the intramolecular electronic transition bands (at 360, 430, and 600 nm) can be seen. Interestingly, these spectral intensities reached a maximum when P ⊥ R (Figure 6 inset). Therefore, the transition dipole moment for CT absorption [parallel to the column axis (*c*-axis)] and that for intramolecular absorptions [parallel to the long axis of TCNQ (mainly *b*-axis)] should be oriented perpendicular to R. In other words, the uniaxial orientation of *a*-axis is induced along with R (Supplementary Figure 5). This rubbing-induced change in molecular alignment is distinct from those observed for round-discoidal columnar liquid crystals (Yoshio et al., 2004; Yasuda et al., 2006), and is rather similar to the behavior of rod-like liquid crystals. The unique structural transformation characteristics would be due primarily to the oval-like molecular structure of DHP derivatives and ICT interactions in the supramolecular copolymers.

**Figure 7:**
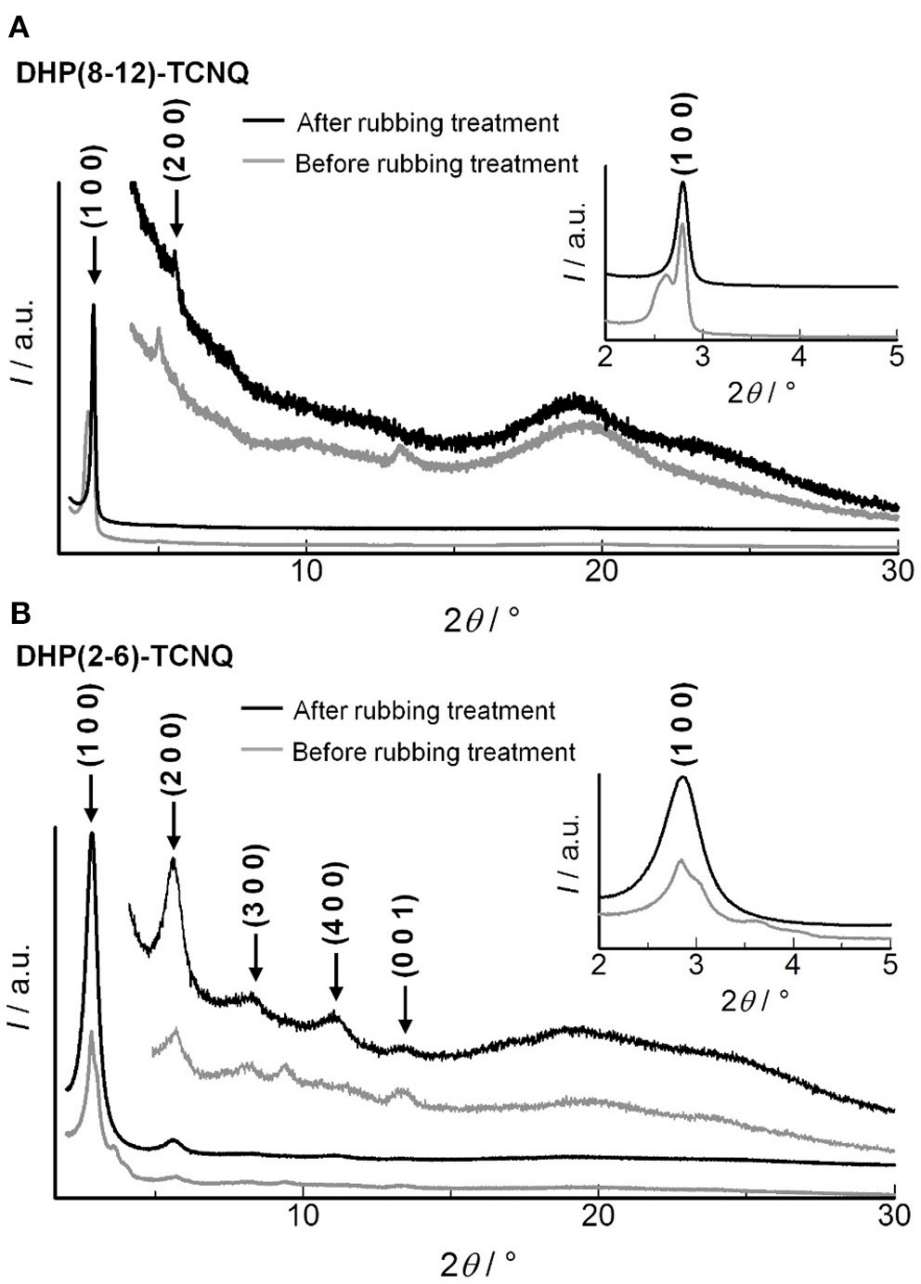
XRD patterns for **(A) DHP(8-12)-TCNQ** and **(B) DHP(2-6)-TCNQ** before (gray) and after (black) the repeated directionally random rubbing treatment. Peaks after the rubbing treatment are assigned, as shown in black indices. Detailed assignments of the peaks before and after the rubbing are shown in Tables 1, 2, respectively.

The shear-force-responsive structural transformation characteristics are also confirmed by changes in the XRD patterns of randomly rubbed films. As shown in Figure 7A, (110) and (200) reflections of Col_r_ phase were fused to a single peak (2θ = 2.79°), and the other peak appeared at 5.58° after the rubbing treatment in **DHP(8-12)-TCNQ**. Since the experimental diffraction spacing (*d*_exp_) of these peaks are 31.6 and 15.8 Å, respectively, Miller indices of (100) and (200) were reasonably assigned to these peaks (Table 2), suggesting that the liquid crystalline phase was changed from Col_r_ phase to a smectic-like mesophase. Such coexistence of columnar and smectic phases has rarely been reported (Nguyen et al., 1990). This mechanical shearing-induced structural change was also observed for the short-chained **DHP(2-6)-TCNQ**, which showed more distinct peaks assignable to (*h*00) reflections (1 ≤ *h* ≤ 4) with the remaining (001) reflection related to the alternate π-stacking after the rubbing treatment (Figure 7B; Table 2). However, the stability of macroscopic alignment of the shear-induced smectic-like phase under the repeated rubbing treatment was inferior to that of **DHP(8-12)-TCNQ** (Supplementary Figures 2D–F, 3). Therefore, the alkyl-chain units assume a large role in determining the inter-column interactions that contribute to the enhanced thermal stability. Longer and more entangled alkyl moiety in **DHP(8-12)-TCNQ** should maintain the macroscopic alignment even after repeated rubbing treatment. Although the long-range ordering along with alternate DA stacking, which is related to the intensity of (001) reflection, seems to get weaker during the structure transformation process, the strong ICT absorption band was maintained without shifting, as shown in Figure 6. It indicates that the alternate DA stacking was retained in the short-range due to the large cohesive energy of ICT complexes. The control on in-plane macroscopic orientations has not been observed for the past ICT complexes while laser-heat-induced dichroism has been reported for neutral CT liquid crystals (Van Winkle et al., 2018). These results enhance the potential of ICT complexes as processable functional electronic materials.

**Table 2:** XRD data for **DHP(8-12)-TCNQ** and **DHP(2-6)-TCNQ** after the rubbing treatment.

	**2θ/^◦^^a^**	***d*_exp_/^b^**	***d*_theo_/^b^**	**Miller index**
**DHP(8**-**12)-TCNQ**	2.79	31.6	31.6	(1 0 0)
Smectic-like phase	5.58	15.8	15.8	(2 0 0)
*a* = 31.6 Å	18.7 br	4.74		alkyl chain
	23–27	3.9–3.3		π − π stacking
**DHP(2**-**6)-TCNQ**	2.86	30.9	31.5	(1 0 0)
Smectic-like phase	5.62	15.7	15.7	(2 0 0)
	8.28	10.7	10.5	(3 0 0)
*a* = 31.5 Å	11.2	7.93	7.87	(4 0 0)
*c* = 6.60 Å	13.4 w	6.6	6.6	(0 0 1)
	18.8 br	4.71		alkyl chain
	23–27	3.9–3.3		π − π stacking

a *Characteristics of the reflections: br, broad; w, weak*.

b *d_exp_ and d_theo_ are the experimentally measured and theoretical diffraction spacings, respectively*.

## Conclusion

The strong π-conjugated electron-donor liquids [**DHP(8-12)** and **DHP(2-6)**] were newly developed. Upon casting the mixed toluene solution of liquid DHP derivatives and TCNQ, alternating supramolecular copolymerization cooperatively occurred into well-defined, alternately stacked ICT complexes linearly aligned in the columnar liquid crystalline phase. The significance of the present work is three-fold. Firstly, the strong liquid donor molecules were developed by covalently accumulating branched alkyl chains to the dihydrophenazine chromophore. Secondly, the ICT liquid crystals are easily obtained simply by casting the equimolar solutions of liquid donors and TCNQ at room temperature. The formation of continuous, alternating supramolecular ICT states in the 1D columns and their organization in liquid crystalline assemblies occurred in the course of dynamic concentration changes (i.e., casting), would be explainable by the presence of cooperativity in the self-assembly process. The ICT interactions occur between the liquid donors and TCNQ molecules, which evolved the distinct Col_r_ liquid crystalline phases with the first clear evidence for the alternate stacking. Thirdly, the columnar liquid crystalline phases of ICT complexes are transformed into the smectic-like mesophase by applying mechanical shear force. The stimuli-responsiveness and macroscopic control in the in-plane molecular alignment of ICT complexes have been achieved for the first time. The emergence of the ICT liquid crystalline phase from alternating supramolecular copolymerization of liquid donors and acceptors discussed in this work gives rise to a new phase-crossover approach (Ishiba et al., 2015), which would be widely applicable to various combinations of π-conjugated liquids and molecular solids. These molecular hybrids would new properties based on the nano-interface effects (Yamamoto et al., 2020), and provide a new platform for developing soft molecular devices with unique electronic characteristics.

## Materials and Methods

Essential experimental procedures are described below. Other details are shown in the Supplementary Material.

### Materials and Instrumentation

TCNQ was purified by sublimation. Toluene was spectroscopic grade (Kishida Chemical). Other commercially available chemicals were reagent grade and used as received. 3,4,5-tris((2-octyldodecyl)oxy)benzoic acid and 3,4,5-tris((2-ethylhexyl)oxy)benzoic acid were synthesized according to the previous reports (Ghosh et al., 2008; Noguchi et al., 2008). Dihydrophenazine derivatives, **DHP(8-12)**, **DHP(2-6)**, and **DHP1**, were synthesized by reactions between 5,10-Bis(3-bromopropyl)-5,10-dihydrophenazine (**DHP(C3Br)**) and 3,4,5-substituted benzoic acids according to Supplementary Scheme 1. The synthetic procedure of **DHP(C3Br)** from phenazine is described in the Supplementary Material. Solid-state UV-Vis-NIR spectra were measured on JASCO V-670 or V-570 (for polarized UV-Vis-NIR spectra with GPH-506 polarizer). DSC was measured on SII DSC-6100. Optical textures were obtained with Nikon ECLIPSE LV100POL. ^1^H NMR spectra were measured on Bruker Avance 300 (300 MHz) spectrometers or Bruker Avance 500 (500 MHz) spectrometers. The ^1^H NMR spectra were calibrated against TMS (δ = 0.0 ppm) or residual internal C_6_D_5_CD_2_H (δ = 2.09 ppm). The redox potential of **DHP(2-6)** was obtained from the cyclic voltammograms acquired by BAS ALS Model 620D. CH_2_Cl_2_ solution containing 1 mM **DHP(2-6)** and 0.1 M TBAClO_4_ was prepared for cyclic voltammetry measurement. Glassy carbon (GC) electrode and Pt wire were used as working electrode and counter electrode, respectively. Reference electrode was Ag/Ag^+^ (0.01 M AgNO_3_, 0.1 M TBAClO_4_ in acetonitrile). The potential was calibrated by using ferrocene as an external standard. Elemental analyses were performed at the Center of Elemental Analysis, Faculty of Science, Kyushu University.

### Syntheses of Donor Molecules

#### Phenazine-5,10-Diylbis(Propane-3,1-Diyl)Bis(3,4,5-Tris((2-Octyldodecyl)Oxy)Benzoate) (DHP(8-12))

3,4,5-Tris((2-octyldodecyl)oxy)benzoic acid (202 mg, 0.200 mmol), bis(3-bromopropyl)-5,10-dihydrophenazine (41.9 mg, 0.099 mmol) and K_2_CO_3_ (32.4 mg, 0.234 mmol) was suspended in DMF (3 mL) and heated at 85°C for 9 h. After cooling to RT, the compound is extracted by hexane (2 mL × 3). Collected hexane layer was washed by water (6 mL) and brine (5 mL) followed by evaporation in vacuo to give purple oil. It was purified by chromatography on neutral alumina (activity V) (hexane, *R*_f_ = 0.15) to afford yellow oil (188.5 mg, 0.082 mmol) (yield: 83.5%). ^1^H NMR (500 MHz, C_6_D_5_CD_3_, TMS, 297 K): δ = 7.69 (s, 4H), 6.65–6.62 (m, 4H), 6.28–6.24 (m, 4H), 4.19 [d, 4H, (-OC*H*_2_-CH(C_8_H_17_)-C_10_H_21_)], 4.16 [t, *J* = 6.0 Hz, 4H, (-N-CH_2_-CH_2_-C*H*_2_-OCO-)], 3.93–3.91 [m, 8H, (-OC*H*_2_-CH(C_8_H_17_)-C_10_H_21_)], 3.32–3.29 [m, 4H, (-N-C*H*_2_-CH_2_-CH_2_-OCO-)], 1.90–1.78 [m, 10H, (-N-CH_2_-C*H*_2_-CH_2_-OCO-), (-OCH_2_-C*H*(C_8_H_17_)-C_10_H_21_)], 1.68–1.22 (m, 192H, alkyl), 0.96–0.88 ppm (m, 36H, -Me); elemental analysis calcd (%) for C_152_H_270_N_2_O_10_: C 79.87, H 11.91, N 1.23; found: C 79.96, H 11.85, N 1.49; *T*_g_ (glass transition temperature): −63°C.

#### Phenazine-5,10-Diylbis(Propane-3,1-Diyl)Bis(3,4,5-Tris((2-Ethylhexyl)oxy)Benzoate) (DHP(2-6))

3,4,5-Tris((2-ethylhexyl)oxy)benzoic acid (101 mg, 0.199 mmol), 5,10-bis(3-bromopropyl)-5,10-dihydrophenazine (41.4 mg, 0.098 mmol), and K_2_CO_3_ (38.5 mg, 0.279 mmol) was suspended in DMF (3 mL) and heated at 85°C for 9 h. After cooling to RT, the compound is extracted by hexane (2 mL × 3). Collected hexane layer was washed by water (6 mL) and brine (5 mL) followed by evaporation in vacuo to give dirty-yellow oil. It was purified by chromatography on neutral alumina (activity V) (hexane, *R*_f_ = 0.09) to afford yellow oil (97.7 mg, 0.077 mmol) (yield: 78.4%). ^1^H NMR (500 MHz, C_6_D_5_CD_3_, TMS, 297 K): δ = 7.65 (s, 4H), 6.62–6.58 (m, 4H), 6.26–6.22 (m, 4H), 4.15 [t, *J* = 6.3 Hz, 4H, (-N-CH_2_-CH_2_-C*H*_2_-OCO-)], 4.11–4.05 [m, 4H, (-OC*H*_2_-CH(Et)-C_4_H_9_)], 3.86–3.80 [m, 8H, (-OC*H*_2_-CH(Et)-C_4_H_9_)], 3.29–3.26 [m, 4H, (-N-C*H*_2_-CH_2_-CH_2_-OCO-)], 1.84–1.24 [m, 58H, (-N-CH_2_-C*H*_2_-CH_2_-OCO-), (-OCH_2_-C*H*(Et)-C_4_H_9_), alkyl], 1.06 (t, *J* = 7.5 Hz, 6H, -Me), 0.97 (t, *J* = 7.0 Hz, 6H, -Me), 0.93–0.90 ppm (m, 24H, -Me); elemental analysis calcd (%) for C_80_H_126_N_2_O_10_: C 75.31, H 9.95, N 2.20; found: C 75.45, H 10.01, N 2.41; *T*_g_ (glass transition temperature): −29°C.

#### Phenazine-5,10-Diylbis(Propane-3,1-diyl)bis(3,4,5-Trimethoxybenzoate) (DHP1)

3,4,5-Trimethoxybenzoic acid (87.2 mg, 0.411 mmol), 5,10-bis(3-bromopropyl)-5,10-dihydrophenazine (86.8 mg, 0.205 mmol), and K_2_CO_3_ (67.3 mg, 0.487 mmol) was suspended in DMF (6 mL) and heated at 85°C for 11 h. After cooling to RT, the solution is poured into cold water (25 mL). Yellow precipitation was filtered, dried and recrystallized from hot acetone to afford pale yellow crystal (95.9 mg, 0.140 mmol) (yield: 68.2%). Because this compound is slightly oxidized to form cation radical in chloroform (and CDCl_3_) solution, the signals assigned to the H atoms in DHP and propylene moieties are broad in the NMR spectrum. ^1^H NMR (300 MHz, CDCl_3_, TMS, 297 K): δ = 7.32 (s, 4H), 6.56 (broad, 4H), 6.36 (broad, 4H), 4.47 [broad, 4H, (-N-CH_2_-CH_2_-C*H*_2_-OCO-)], 3.92 [s, 6H, (-OMe)], 3.91 [s, 12H, (-OMe)], 3.65 [broad, 4H, (-N-C*H*_2_-CH_2_-CH_2_-OCO-)], 2.18 [broad, 4H, (-N-CH_2_-C*H*_2_-CH_2_-OCO-]; elemental analysis calcd (%) for C_38_H_42_N_2_O_10_: C 66.46, H 6.16, N 4.08; found: C 66.65, H 5.97, N 4.14.

### Preparation of Liquid Crystalline ICT Complexes

2 mM **DHP(8-12)** or **DHP(2-6)** in toluene was mixed with an equal amount of 2 mM TCNQ in toluene. 500 μL of resulting deep green CT-complex solution was dropped onto the watch grass and dried by natural evaporation. The deep blue residue was redissolved in 50 μL of the CT-complex solution and transferred to the substrate for measurement. Slow evaporation of toluene for ~5 min produced the homogeneous liquid crystalline film. Note that TCNQ may be precipitated during extremely slow evaporation because of the lower solubility of TCNQ in toluene.

### Preparation of Single Crystal of DHP1-TCNQ·2CH_3_CN

Black single crystals of **DHP1-TCNQ·2CH**_**3**_**CN** were obtained by slow diffusion of the following layered solution in a glass tube. Bottom layer: CH_2_Cl_2_ solution of **DHP1** (10 mM, 0.1 mL). Middle layer: 1:1 mixed solvent of CH_2_Cl_2_ and acetonitrile (0.1 mL). Upper layer: acetonitrile solution of TCNQ (2 mM, 0.5 mL).

### X-Ray Powder Diffraction Measurement

The cast film of CT complex was fabricated on Si(100) wafer. X-ray powder diffraction (XRD) patterns were collected using an X-ray diffractometer, SmartLab (Rigaku), in the 2θ scan range of 2–50° using Cu Kα (λ = 0.15406 nm) radiation operated at 30 mA and 40 kV at RT. Sampling and scan widths were 0.01 and 0.1°/min, respectively.

### Single-Crystal X-Ray Structural Analysis

A single crystal of **DHP1-TCNQ·2CH**_**3**_**CN** (size: 0.20 × 0.05 × 0.02 mm) was mounted on a glass tube with grease. Single-crystal X-ray data were collected on a CCD diffractometer (Rigaku Saturn VariMax) with graphite-monochromated Mo Kα radiation (λ = 0.71075 Å). The temperature was set at 293 K by using a Japan Thermal Engineering temperature controller. Diffraction intensities were integrated by using CrystalClear software (Rigaku). The crystal structures were solved by using direct methods [SHELXS-97 (Sheldrick, 2008)], followed by Fourier syntheses. Structure refinement was performed by using full-matrix least-squares procedures using SHELXL (Sheldrick, 2015) on *F*^2^ in the Yadokari-XG 2009 software (Wakita, 2001; Kabuto et al., 2009).

### Crystallographic Data for DHP1-TCNQ·2CH_3_CN

CCDC-2056279 contains the supplementary crystallographic data for **DHP1-TCNQ·2CH**_**3**_**CN**. It can be obtained free of charge from The Cambridge Crystallographic Data Centre via www.ccdc.cam.ac.uk/data_request/cif.

## Data Availability Statement

The original contributions presented in the study are included in the article/Supplementary Material, further inquiries can be directed to the corresponding authors.

## Author Contributions

HI mainly executed the conceptualization, syntheses, and measurement. HF contributed to the syntheses of 3,4,5-substituted benzoic acids. NK supervised this work and carried out discussions. HI and NK co-wrote the paper. All authors contributed to the article and approved the submitted version.

## Conflict of Interest

The authors declare that the research was conducted in the absence of any commercial or financial relationships that could be construed as a potential conflict of interest.

## Supplementary Material

Additional synthetic procedures, results on rubbing treatment, crystal structure information, spectroscopic data, cyclic voltammogram, calculation of the degree of CT (ρ_CT_), and DSC thermograms are described.
