# Supplementary material for: Ionic Charge-Transfer Liquid Crystals Formed by Alternating Supramolecular Copolymerization of Liquid π-Donors and TCNQ
Source: Front Chem. 2021 Mar 29;9:657246. doi: 10.3389/fchem.2021.657246 (PMC8039295; doi:10.3389/fchem.2021.657246)
Supplement: Supplementary file 1 [file Table_1.docx]

Supplementary Material

# Supplementary Data

## Synthetic procedures

### 5,10-Dihydrophenazine (DHP(H))

**DHP(H)** was prepared by previously reported procedures (Sugimoto et al., 1989; Holzapfel et al., 2002). A solution of sodium dithionite (14.32 g, 82.3 mmol) in water (130 mL) was added to a boiling solution of phenazine (1.08 g, 6.01 mmol) in EtOH (30 mL). The pale yellow suspension was refluxed for 2 h. The precipitate was filtered off, washed by water and dried over phosphorus pentoxide (P_4_O_10_) under reduced pressure. DHP(H) was obtained as air-sensitive greenish gray solid, which was used without further purification. (1.07 g, 5.85 mmol) (yield: 97.4%) IR (ATR): *ν* 3379, 1612, 1467, 1452, 1298, 1124, 1038, 908 cm^–1^.

### 5,10-Bis(3-bromopropyl)-5,10-dihydrophenazine (DHP(C3Br))

**DHP(C3Br)** was prepared by adapting a procedure for the synthesis of 5,10-dihydro-5,10-dimethylphenazine (Girl et al., 2001). The suspension of Na_2_CO_3_ (452 mg, 4.26 mmol), Na_2_S_2_O_4_ (500 mg, 2.87 mmol), tetrabutylammonium bromide (229 mg, 0.71 mmol), DHP(H) (275 mg, 1.51 mmol) and 1,3-dibromopropane (1.66 g, 8.25 mmol) in acetonitrile (3 mL) and water (1.6 mL) was heated at 85 °C for 10 h under nitrogen atmosphere. The mixture was poured into 33 mL of water, and extracted by toluene (20 mL ×2). The organic layer was dried over K_2_CO_3_ and evaporated at 90 °C in vacuo to give deep purple oil. It was purified by chromatography on neutral alumina (lower filler: activity V, upper filler: activity I) (toluene : hexane = 1:4, R_f_ = 0.67) followed by recrystallization from hexane. Light-sensitive yellow-green crystals were obtained. (107 mg, 0.25 mmol) (yield: 16.7%). ^1^H NMR (300 MHz, C_6_D_5_CD_3_, residual internal C_6_D_5_CD_2_H (*δ* 2.09), 297 K): *δ* 6.61–6.55 (m, 4H), 6.17–6.11 (m, 4H), 3.20–3.15 (m, 4H), 2.83 (t, *J* = 6.2 Hz, 4H), 1.73–1.64 (m, 4H); elemental analysis calcd (%) for C_18_H_20_Br_2_N_2_: C 50.97, H 4.75, N 6.60; found: C 51.38, H 4.83, N 6.68.

# Supplementary Scheme

**Supplementary Scheme 1.** Synthesis of **DHP(8-12)**, **DHP(2-6)** and **DHP1**.

# Supplementary Figures

## Cyclic voltammogram of DHP(2-6)

**Supplementary Figure 1.** Cyclic voltammogram of 1 mM **DHP(2-6)** in CH_2_Cl_2_ (0.1 M TBAClO_4_) at a GC electrode. Scan rate = 100 mV s^–1^.

## Changes in macroscopic alignment of DHP(2-6)-TCNQ induced by multiple rubbing treatment

Strong inter-column interactions along the long axis of **DHP(8-12)-TCNQ** would be derived from mutual penetration and entanglement of alkyl chains between the neighbouring columns. They play a key role in stabilizing the shear-induced macroscopic alignment of **DHP(8-12)-TCNQ** liquid crystal, as indicated by the following experiments.

In the case of ICT liquid crystal formed from shorter-chained liquid donor **DHP(2-6)** and TCNQ, in-plane anisotropy was similarly observed for **DHP(8-12)-TCNQ** liquid crystal after the first rubbing treatment (Supplementary Figures 2a-c). Meanwhile, upon repeating the rubbing treatment, the in-plane anisotropy was lost, as shown in Supplementary Figures 2d-f. The loss of anisotropy is also observed in the polarized absorption spectra (Supplementary Figure 3) of the repeatedly rubbed film of **DHP(2-6)-TCNQ**. However, the XRD data (Figure 7, Table 2) shows that the smectic-like liquid-crystalline phase remained even after repeated rubbing treatment. These results indicate that the macroscopic alignment of the liquid crystal obtained by a single rubbing treatment was turned into microscopic smectic-like domains by the repeated rubbing treatment. The observed fragmentation by excessive rubbing treatment would reflect weaker inter-column interactions available for the ICT liquid crystals of short-chained **DHP(2-6)-TCNQ**.


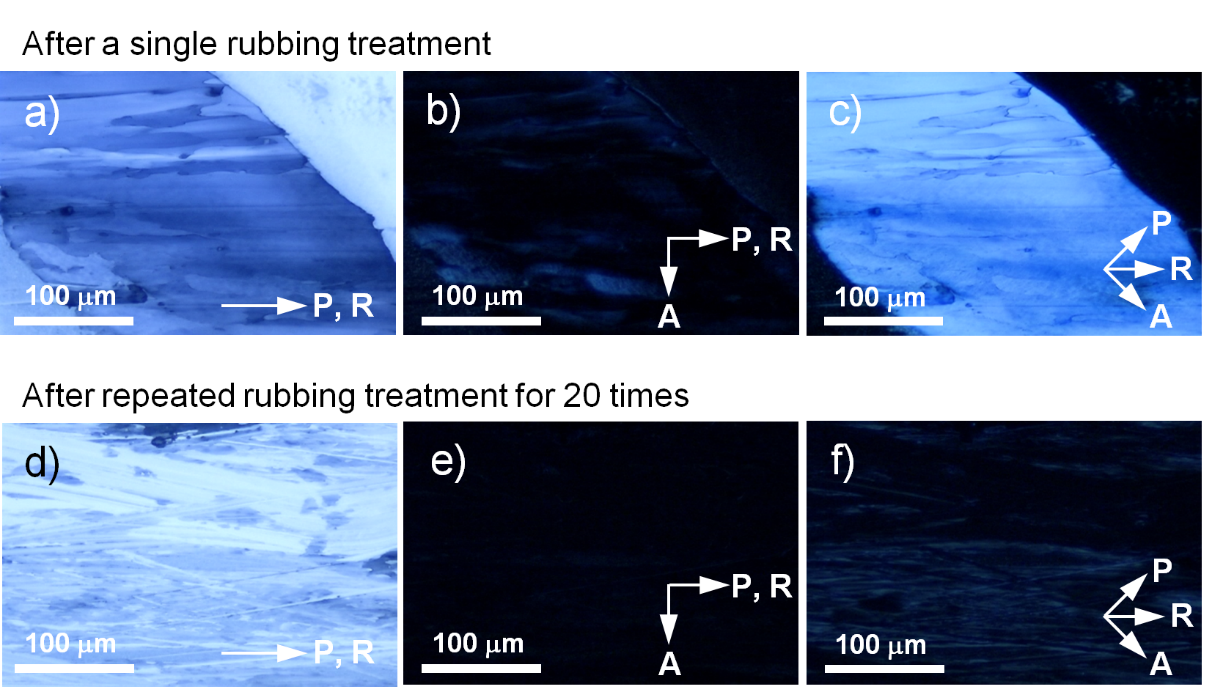


**Supplementary Figure 2.** Polarized optical micrographs of the films of **DHP(2-6)-TCNQ**. a-c) after a single rubbing treatment, and d-f) after repeated rubbing treatments 20 times. a,d). Photographs taken without an analyzer. The polarizer axis is parallel to the rubbing direction (R). b,e) Photographs taken under crossed polarizers. The polarizer axis is parallel to the rubbing direction. c,f) Photographs taken under crossed polarizers. The polarizer axis is fixed at a 45° angle from the rubbing direction.

**Supplementary Figure 3.** Variation of polarized UV-Vis-NIR spectra of the repeatedly rubbed film of **DHP(2-6)-TCNQ** with increasing the angle between polarizer (P) and rubbing (R) direction in steps of 30°.

## IR spectrum of DHP1-TCNQ•2CH3CN


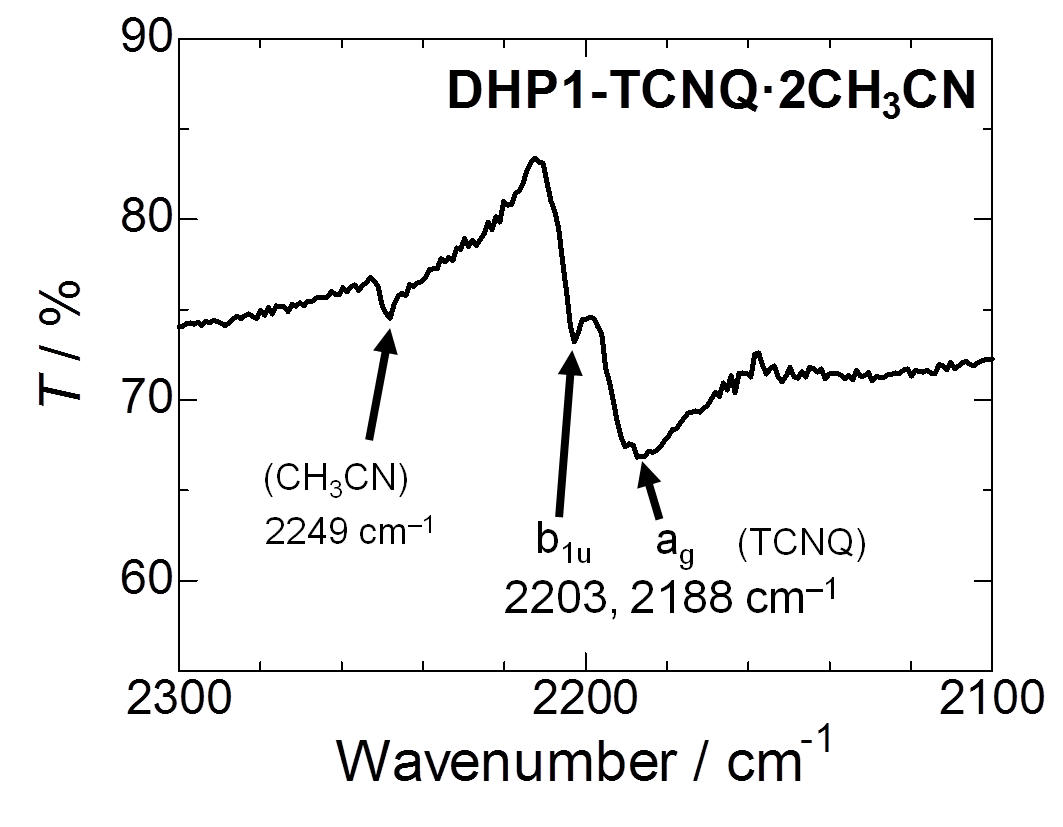


**Supplementary Figure 4.** IR spectra of **DHP1-TCNQ•2CH_3_CN** with wavenumber value for C≡N stretching vibration (*ν*_C≡N_).

## Calculation of the degree of CT (*ρ*_CT_) via the frequency of C≡N stretching vibration (*ν*_C≡N_)

The linear correlation between the charge of TCNQ and the frequency of its C≡N stretching vibration (*ν*_C≡N_) was reported by Chappell et al. in 1981. In this work, a neutral TCNQ (*ρ*_CT_ = 0) gave the peak of *ν*_C≡N_ at 2222 cm^–1^. K-TCNQ (*ρ*_CT_ = 1) was reported to give a *ν*_C≡N_ peak at 2183 cm^–1^. Therefore, the following equation can be used to estimate the charge of TCNQ molecule.

$$\rho_{\mathrm{CT}}=\frac{2222-x}{2222-2183}=\frac{2222-x}{39} (eq.1)$$

where *x* is the frequency of *ν*_C≡N_ (cm^–1^) in the target TCNQ molecule. Assigning the values (2197 cm^–1^ for **DHP(8-12)-TCNQ** and 2195 cm^–1^ for **DHP(2-6)-TCNQ**) to (eq.1), the *ρ*_CT_ values for **DHP(8-12)-TCNQ** and **DHP(2-6)-TCNQ** are calculated as 0.64 and 0.69, respectively.

## Schematic representation of the molecular arrangement and transition dipole moment of DHP(8-12)-TCNQ after rubbing treatment


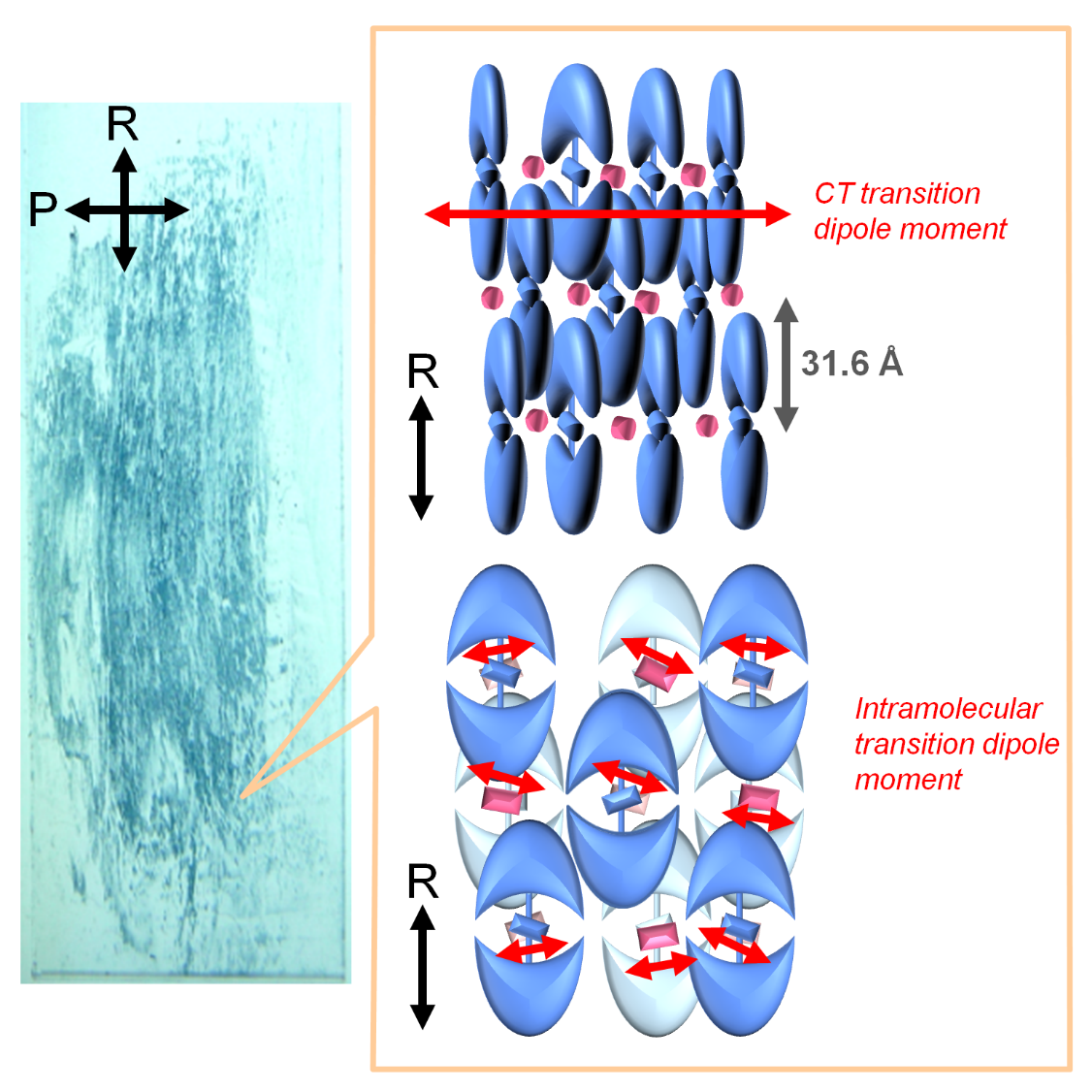


**Supplementary Figure 5.** (Left) Transmission images of the film taken in P ⊥ R. (Right) Schematic representation of the molecular arrangement after the rubbing treatment. The uniaxial orientation of long axis of **DHP(8-12)** induced along R enables both CT absorption and intramolecular absorption perpendicular to R.

## DSC thermograms and glass transition temperature (*T*_g_) of π-donor liquids and ICT liquid crystals


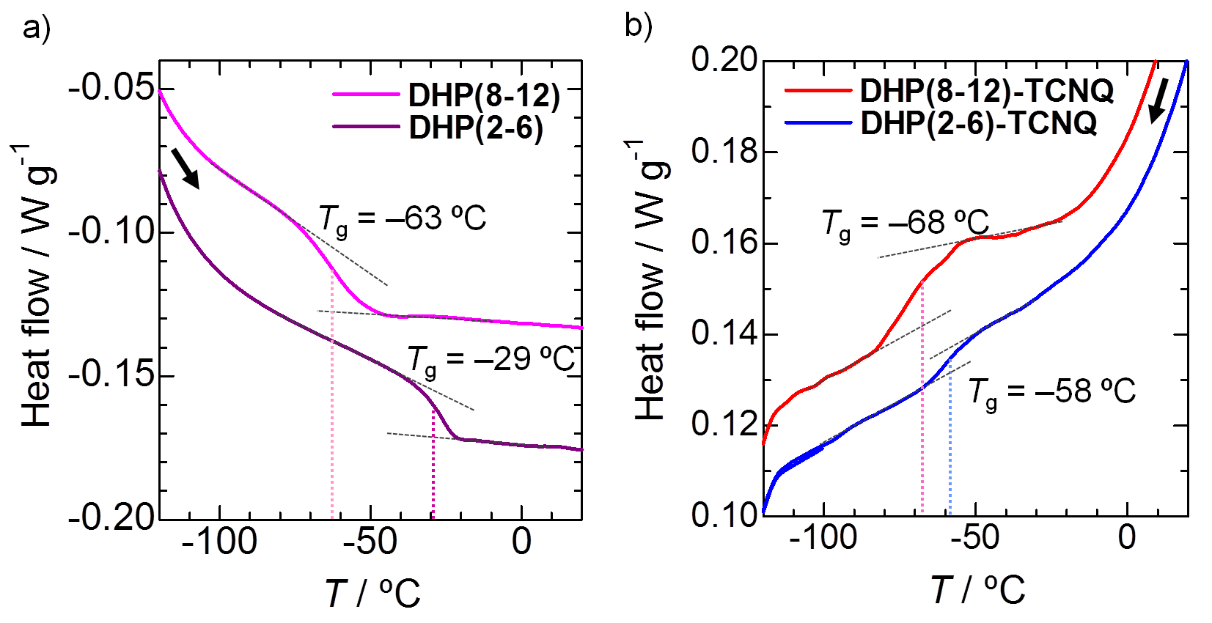


**Supplementary Figure 6.**  DSC thermograms of a) **DHP(2-6)** and **DHP(8-12)**, b) **DHP(2-6)-TCNQ** and **DHP(8-12)-TCNQ**. Glass transition temperatures *T*_g_ are indicated by solid lines (inflection point). We note the *T*_g_ values observed for DHP-TCNQ liquid crystals are comparable or lower than the corresponding liquid DHP molecules. The observed trend of higher *T*_g_ values for pristine liquid DHP molecules would be related to their intrinsic higher configurational entropy by considering statistical thermodynamics.

## Thermal stability of the ICT columnar complex

Upon heating, ICT liquid crystals **DHP(8-12)-TCNQ** and **DHP(2-6)-TCNQ** showed melting above 80 ºC, which was accompanied by segregation of TCNQ crystals as shown in Supplementary Figure 7. Further heating of the mixture gave isotropic liquid over 120 ºC. These heat-induced phase-separated samples naturally did not return to the original liquid-crystalline ICT phase anymore. The thermal characteristics of **DHP(8-12)-TCNQ** and **DHP(2-6)-TCNQ** and their irreversible structural change were investigated by DSC measurement. Supplementary Figure 8 shows DSC thermograms obtained for **DHP(8-12)-TCNQ** and **DHP(2-6)-TCNQ**, respectively. Upon heating, they show endothermic peaks around 85 °C and exothermic peaks around 120 °C. These peaks are not observed in the second scan (red lines), indicative of the decomposition of CT complexes and phase separation of solid TCNQ at these elevated temperatures. The CT absorption also disappeared in the resultant isotropic liquid, as shown in Supplementary Figure 9. This irreversible melting was also observed for previous columnar liquid-crystalline ICT complexes (Saeva et al., 1982; Stępień et al., 2007).


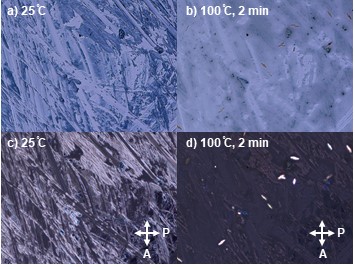


**Supplementary Figure 7.** Optical micrographs of the randomly scratched cast film of **DHP(8-12)-TCNQ** taken at 25 °C (a,c) and taken after keeping 2 minutes at 100 °C (b,d). Photographs of b,d) were taken under crossed polarizers.


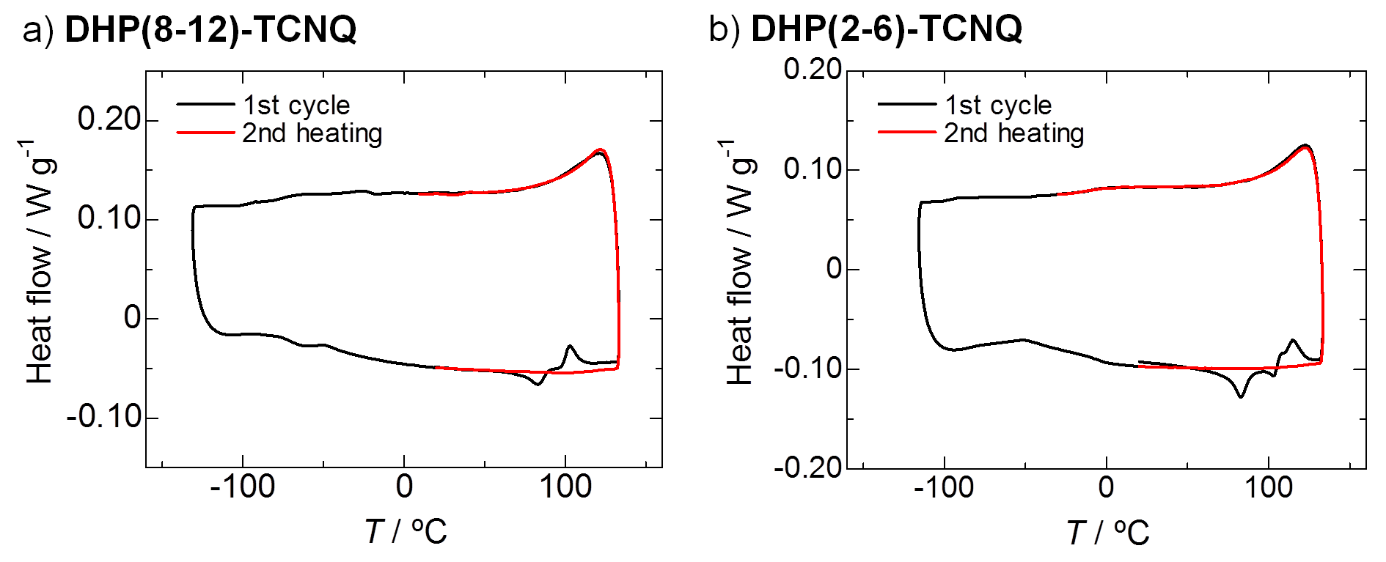


**Supplementary Figure 8.** DSC thermograms of a) **DHP(8-12)-TCNQ** and b) **DHP(2-6)-TCNQ**.

**Supplementary Figure 9.** UV-Vis-NIR spectra of **DHP(8-12)-TCNQ** in the initial ICT liquid-crystalline state (black) and in isotropic liquid obtained after heating (red). Temperature, 25 °C.

## ^1^H NMR spectra


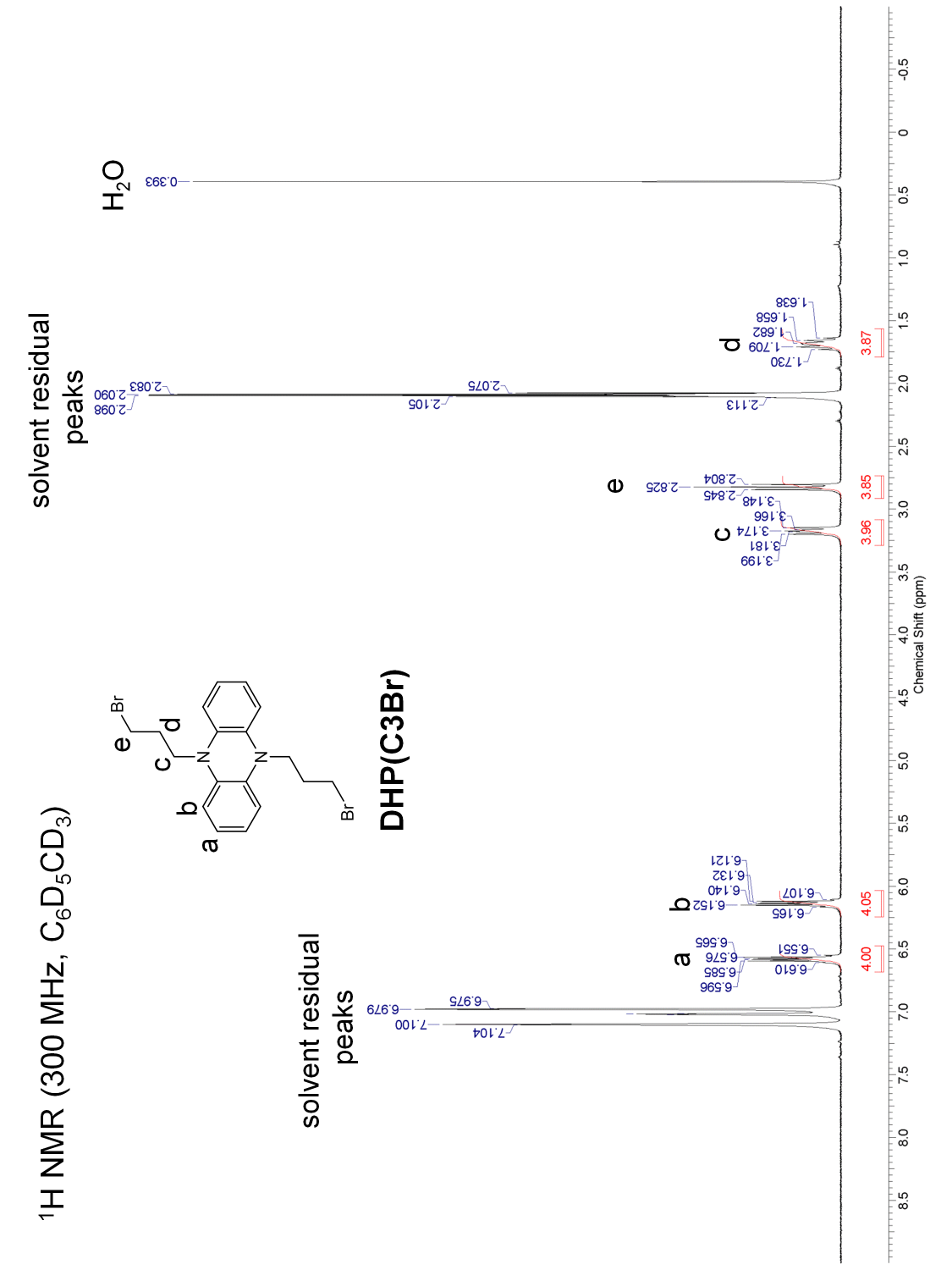


**Supplementary Figure 10.** ^1^H NMR spectrum of **DHP(C3Br)**


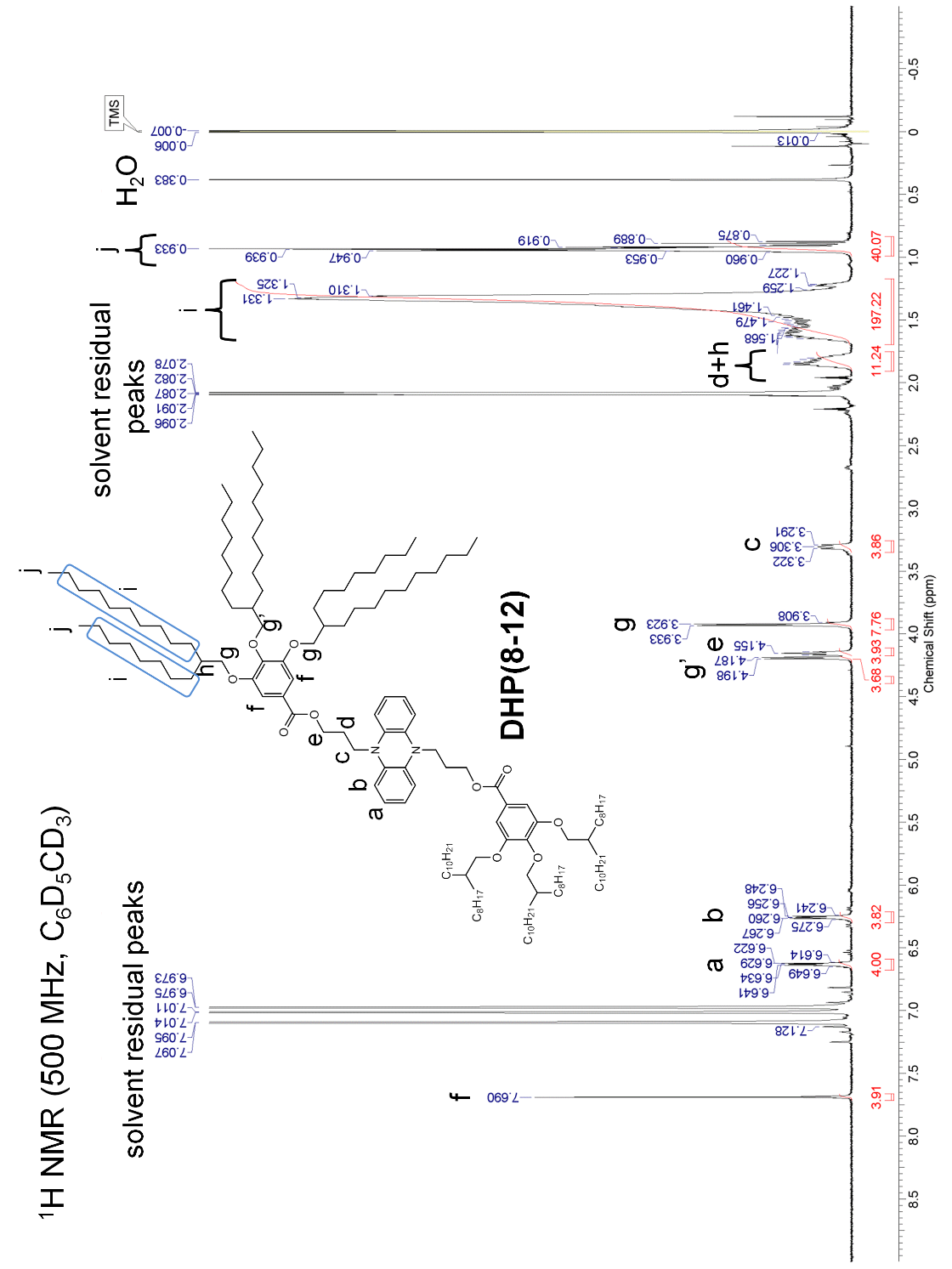


**Supplementary Figure 11.** ^1^H NMR spectrum of **DHP(8-12)**


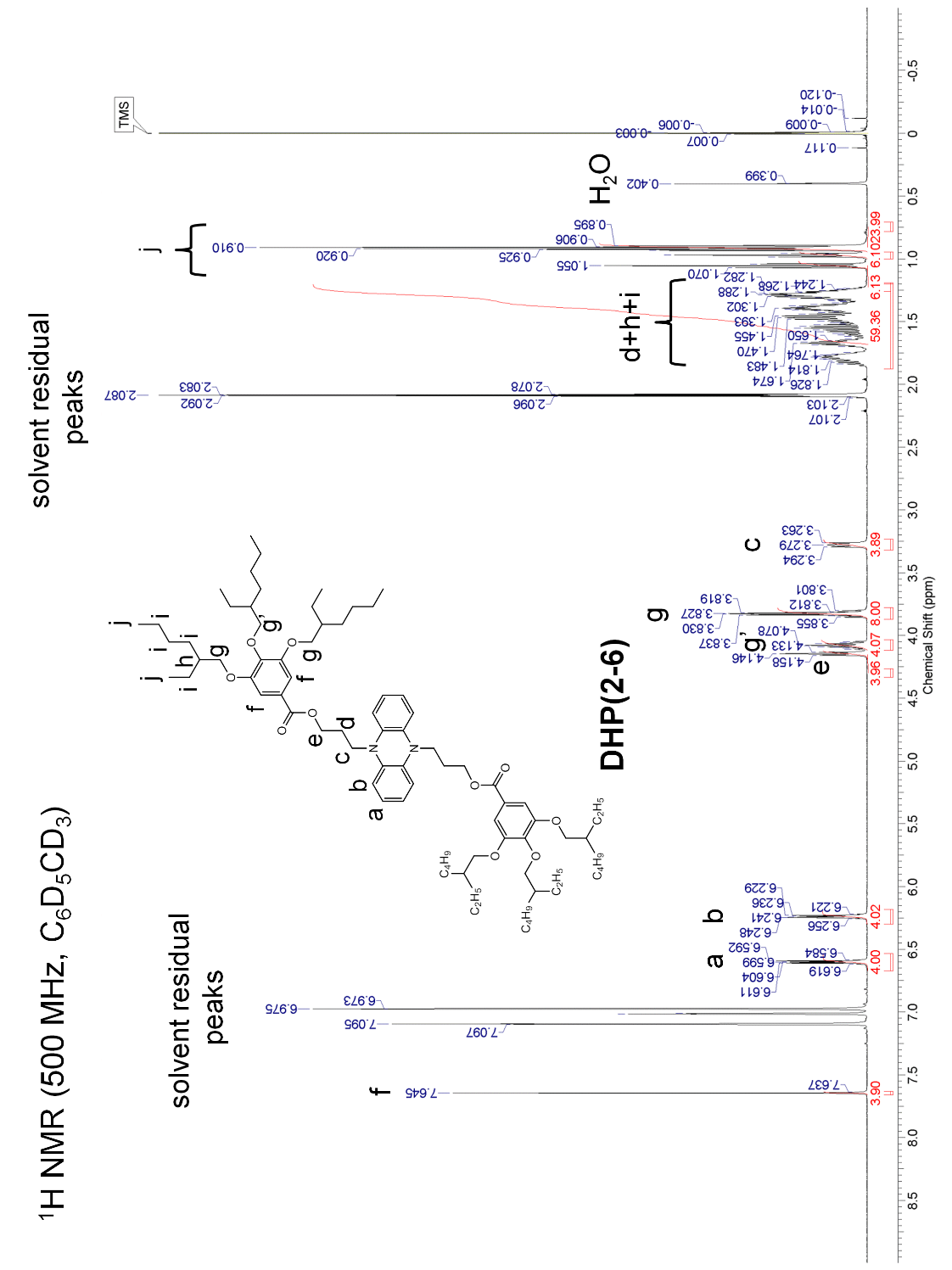


**Supplementary Figure 12.** ^1^H NMR spectrum of **DHP(2-6)**


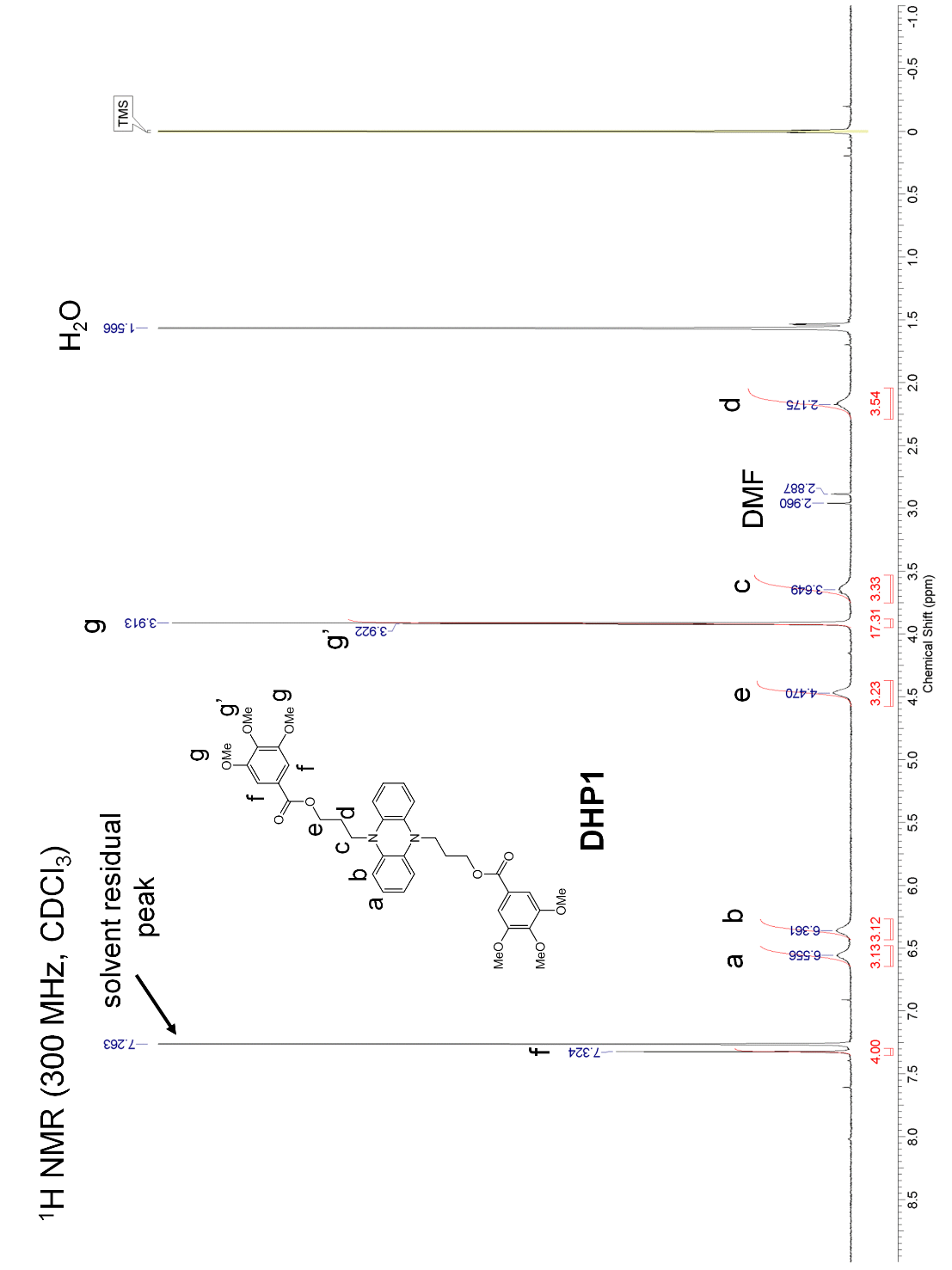


**Supplementary Figure 13.** ^1^H NMR spectrum of **DHP1**

# Supplementary Tables

**Table S1.** Crystallographic data and structural refinement parameters for **DHP1-TCNQ•2CH_3_CN**.

| Empirical formula | C_54_H_52_N_8_O_10_ |
| --- | --- |
| Formula weight | 973.04 |
| Crystal size (mm) | 0.20 × 0.05 × 0.02 |
| Temperature (K) | 293 |
| Crystal system | Triclinic |
| Space group | *P*-1 |
| *a* (Å) | 7.0306(14) |
| *b* (Å) | 17.342(3) |
| *c* (Å) | 21.155(4) |
| *α* (°) | 104.770(9) |
| *β* (°) | 92.646(10) |
| *γ* (°) | 101.482(13) |
| Volume (Å^3^) | 2431.4(8) |
| *Z* | 2 |
| *D*(calc.) (g cm^-3^) | 1.329 |
| *μ*(Mo-Kα) (mm^-1^) | 0.093 |
| *F*(000) | 1024 |
| Theta range (º) | 3.00–27.47 |
| Total reflections | 19730 |
| Unique reflections | 10686 |
| Goodness of fit | 0.908 |
| *R, wR_2_* [*I* > 2*σ*(*I*)] | 0.0675, 0.1398 |
| *R, wR_2_* (all data) | 0.2157, 0.1977 |

**Table S2.** Distance between atoms in **DHP1** and TCNQ plane in the crystal structure of **DHP1-TCNQ•2CH_3_CN**.

| Atoms in **DHP1** | Distance to planes / Å | |
| --- | --- | --- |
|  | TCNQ | TCNQ_#1 |
| N1 | 3.443(4) | 3.328(3) |
| N2 | 3.173(3) | 3.598(4) |
| C1 | 3.303(3) | 3.337(3) |
| C6 | 3.221(3) | 3.550(4) |
| C7 | 3.334(4) | 3.437(3) |
| C12 | 3.434(4) | 3.337(3) |
|  |  |  |
| **Average** | **3.318** | **3.431** |

Symmetry transformations used to generate equivalent atoms: #1: *x*+1, *y*, *z*.

# Supplementary References

Girl, P., Byker, H. J., and Baumann, K. L. (2001). One pot synthesis of 5,10-dihydrophenazine compounds and 5,10-substituted dihydrophenazines. U.S. PatentNo 6,242,602. Washington, DC: U.S. Patent and Trademark Office.

Holzapfel, M., Lambert, C., Selinka, C., and Stalke, D. (2002). *J. Chem. Soc. Perkin Trans. 2* 1553–1561. doi: 10.1039/B204392K

Saeva, F. D., Reynolds, G. A., and Kaszczuk, L. (1982). Liquid-crystalline cation-radical charge-transfer systems. *J. Am. Chem. Soc.* 104, 3524–3525. doi: 10.1021/ja00376a055

Stępień, M., Donnio, B., and Sessler, J. L. (2007). Discotic Liquid-Crystalline Materials Based on Porphycenes: A Mesogenic Metalloporphycene–Tetracyanoquinodimethane (TCNQ) Adduct. *Chem. Eur. J.* 13, 6853–6863. doi: 10.1002/chem.200700125

Sugimoto, A., Kotani, T., Tsujimoto, J., and Yoneda, S. (1989). Preparation and properties of electron donor acceptor complexes of the compounds having capto-dative substituents. *J. Heterocycl. Chem.* 26, 435–438. doi: 10.1002/jhet.5570260232
